# Cerevisterol from *Ophiocordyceps sinensis* fruiting bodies against liver fibrosis

**DOI:** 10.3389/fphar.2026.1825109

**Published:** 2026-07-08

**Authors:** Minting Yang, Tao Sun, Richou Han, Huichun Xie, Li Cao

**Affiliations:** 1 Qinghai Normal University, Xining, China; 2 Guangdong Key Laboratory of Animal Conservation and Resource Utilization, Guangdong Public Laboratory of Wild Animal Conservation and Utilization, Institute of Zoology, Guangdong Academy of Sciences, Guangzhou, China

**Keywords:** cerevisterol, liver fibrosis, LX-2 cells, Ophiocordyceps sinensis, TGF-β1

## Abstract

**Objective:**

Liver fibrosis is a critical pathological process to irreversible cirrhosis. The valued *Ophiocordyceps sinensis* (Berk.) G.H. Sung, J.M. Sung, Hywel-Jones and Spatafora (Ophiocordycipitaceae, Hypocreales) fungus exhibits remarkable hepatoprotective effects, especially anti-inflammation. However, detailed metabolites with anti-inflammation activity need to be explored from this fungus. This study investigates the anti-liver fibrosis effect of cerevisterol extracted from *O. sinensis* fruiting bodies on TGF-β1-induced LX-2 cells (a human hepatic stellate cell line) and the underlying potential mechanisms.

**Methods:**

The metabolites were extracted and purified by petroleum ether, ethanol reflux extraction and chromatographic separation technology. Their structures were identified based on extensive NMR and MS data. TGF-β1-induced LX-2 cells were employed to study the anti-inflammation activity of the isolated cerevisterol. The mRNA expression of *Smad3, Smad4*, and *Collagen I* in the cells was verified by qRT-PCR and Smad3 and Smad4 proteins by Western blot.

**Results:**

Cerevisterol, adenosine, uridine, ergosterol, 2-piperidone, L-tryptophan and (24S)-ergost-7(8)-en-3β,5α,6β-triol were obtained. In the TGF-β1-induced fibrosis model, cerevisterol (10 μM) and a commercial anti-liver fibrosis drug containing Chinese cordyceps (75 ng/mL) as positive control significantly suppressed cell proliferation at 72 and 96 hours. qRT-PCR analysis demonstrated that cerevisterol downregulated mRNA expression of Smad3, Smad4, and Collagen I. Western blot results confirmed reduced protein levels of Smad3 and Smad4 in the cerevisterol-treated cells.

**Discussion:**

Cerevisterol from *O. sinensis* fungus exerts anti-liver fibrosis effects by inhibiting hepatic stellate cell activation and proliferation, and by downregulating TGF-β/Smad3 signaling pathway, thereby reducing excessive extracellular matrix-related gene and protein expression.

## 1 Introduction

Liver fibrosis is a pathological and physiological process characterized by the excessive and diffuse deposition of extracellular matrix (ECM) in the liver, mainly collagen, leading to interstitial fibrosis (Lemoinne and Friedman, 2019). If the damaging factors cannot be removed for a long time, the fibrosis process will persist and eventually develop into liver cirrhosis (Du et al., 2025). Hepatic stellate cells (HSC) play a key role in the process of liver fibrosis. Activated HSCs transform into myofibroblast-like cells (MFC) and fibroblasts, which is the core link in the occurrence and development of fibrosis (Tsuchida and Friedman, 2017). Liver fibrosis is induced by many factors, including transforming growth factor-β (TGF-β) (Akkız et al., 2024; Xu et al., 2016). The search for hepatocyte-protective drugs becomes a key target for the treatment of liver fibrosis.

The TGF-β/Smad3 signaling pathway, a very important route in the cell signaling transmission system, plays a crucial role in regulating cell proliferation, differentiation, apoptosis, immune response, and the generation of ECM, especially in the occurrence and development of fibrosis and tumors (Giarratana et al., 2024). TGF-β is a multifunctional cytokine family, including TGF-β1, TGF-β2, and TGF-β3, which bind to the receptors on the cell surface, activate downstream Smad proteins, and ultimately regulate gene expression (Massagué and Wotton, 2020). In liver fibrosis, the TGF-β/Smad3 pathway activates HSCs and promotes the generation of collagen and other ECM metabolites (Ghosh-Roy et al., 2025).

Many anti-hepatic fibrosis drug studies have been conducted in non-alcoholic steatohepatitis and cholestatic diseases in clinical trials, targeting different steps of the pathophysiology of chronic liver injury, including liver metabolism, inflammation and apoptosis, fibrosis and remodeling (Lemoinne and Friedman, 2019).

Although long consumption history of Chinese cordyceps is recorded, this *Ophiocordyceps sinensis* (Berk.) G.H. Sung, J.M. Sung, Hywel-Jones and Spatafora (Ophiocordycipitaceae, Hypocreales) fungus-*Thitarodes* insect complex gained a worldwide attention in the year of summer Olympic game in 1993, as several Chinese laureates used it in their diet (Chen et al., 2013). The mycelia of this fungus exhibits remarkable hepatoprotective effects, especially anti-inflammatory (Cheng et al., 2014) and anti-fibrosis (Liu and Shen, 2003). *O. sinensis* mycelia polysaccharides can activate the mouseRAW264.7 macrophage cell and modulate its cytokine secretion under lipopolysaccharide induction (Liu et al., 2021). *O. sinensis* mycelia protect liver sinusoidal endothelial cells in lipopolysaccharide/D-galactosamine-induced mice, and its protective mechanism was involved in modulation of matrix metalloproteinases (MMP) 22/9 activities and anti-oxidative capability (Peng et al., 2014a). Water extracts of *O. sinensis* mycelia could ameliorate liver acute damage induced by carbon tetrachloride (CCl4) in rats. Furthermore, the anti-fibrotic effect of *O. sinensis* mycelia is associated with its downregulation on the HSC activation (Peng et al., 2014b). The downregulations of TGF-b pathways and NFkB may contribute to protective effect of thioacetamide-induced liver inflammation/fibrosis by *O. sinensis* mycelia (Wu et al., 2017). A traditional Chinese medicine formula composing of *Salvia Miltiorrhiza*, *Prunus davidiana*, cultured *O. sinensis* mycelia, *Schisandra chinensis*, *Pinus massoniana*, and *Gynostemma pentaphyllum*, were used to alleviate liver fibrosis by inhibiting the Notch signaling pathway (Fu et al., 2021). Four polysaccharide fractions obtained from *O. sinensis* mycelia attenuated hepatic bile duct fibrosis by inhibiting the activation of the TGF-β/Smad signaling pathway (Peng et al., 2013). So far, polysaccharides are regarded as the Chinese cordyceps-derived bioactive metabolites for hepatoprotective effects. More molecules with hepatoprotective effects are explored from the Chinese cordyceps.

In this study, the anti-liver fibrosis effect of cerevisterol identified from *O. sinensis* fruiting bodies was demonstrated, via TGF-β1-induced activation of LX-2 cells and the TGF-β/Smad3 signaling pathway, which provides a great potential by using *O. sinensis*-derived metabolite for the anti-inflammatory of liver fibrosis.

## 2 Methods and materials

### 2.1 Cultivation of *O. sinensis* fruiting bodies

*O. sinensis* fruiting bodies (KD1223) was cultured in a rice-wheat based medium using the previously described method, and molecular identification was performed by sequencing the ITS1-5.8S-ITS2 ribosomal DNA region (Cao et al., 2015), in the Institute of Zoology, Guangdong Academy of Sciences, Guangzhou, China. Briefly, the flasks containing 100 mL PPDA liquid medium (potato dextrose liquid medium supplemented with 10% peptone) with *O. sinensis* blastospores and mycelia were incubated at 13 °C on a 100-rpm shaker for 50 days. After diluted 3 times with sterile water, 12 mL of the fungal culture in the flasks were introduced into the sterilized rice medium containing 16 g of rice, 0.4 g of silkworm pupae powder, and 20 mL of nutrient solution (20 g glucose, 2 g KH_2_PO_4_, 1 g MgSO_4_, 1 g ammonium citrate, 5 g peptone, 20 mg vitamin B_1_, and 1000 mL distilled water) in a glass bottle (diameter, 50 mm; height, 90 mm). The glass bottles were incubated in the dark at 9 °C-13 °C for 50 days, followed by cold induction at 4 °C for about 100 days, and kept at 13 °C for 40 days. The fruiting bodies were harvested for the extraction of bioactive metabolites.

### 2.2 Extraction, purification and identification of the metabolites

Ultrasonic-assisted extraction combined with reflux extraction was adopted (Akbal et al., 2024). Dried powder of *O. sinensis* fruiting bodies was crushed and sieved through a 40-mesh sieve to obtain homogeneous powder, which was vacuum-dried at 60 °C to constant weight. Petroleum ether (60 °C-90 °C) was used for reflux extraction of the powder 3 times (2 h each time) to remove lipophilic impurities such as lipids and pigments (Ahmed et al., 2018). After the residue was air-dried, 95% ethanol was applied for ultrasonic extraction 3 times (200W power; temperature at 40 °C; 1 h each time). The ethanol extract was collected and concentrated under reduced pressure (vacuum degree 0.08MPa, temperature 50 °C) to obtain ethanol extract paste (Chen et al., 2022). The paste was suspended in an appropriate amount of distilled water, followed by sequential fractional extraction with ethyl acetate and n-butanol (extraction ratio 1:1, repeated 3 times). The ethyl acetate fraction, n-butanol fraction, and aqueous fraction were collected separately and concentrated under reduced pressure to obtain extracts of three fractions with different polarities (Lautens and Johnson, 2015).

Column chromatography was used for first step separation to split complex metabolites. Silica gel G (200-300 mesh) was used with gradient elution by petroleum ether-ethyl acetate and chloroform-methanol (Zhao et al., 2022). The polarity was gradually adjusted from low to high (petroleum ether: ethyl acetate = 100:0 to 0:100, chloroform: methanol = 100:0 to 0:100). Elution was carried out with eluents, and fractions were collected every 10-20 mL. Thin-layer chromatography (TLC) was used for tracking and detection. Fractions with the same Rf value were combined to obtain multiple purified metabolites (Mojsiewicz-Pieńkowska, 2008). For larger metabolites obtained from silica gel column separation, Sephadex LH-20 gel column was used for further subdivision. Methanol or chloroform-methanol (1:1) was used as eluent, and metabolites were separated according to molecular weight based on the molecular sieve effect of the gel. The fraction with a single peak was collected for further experiments (Sundarraj et al., 2018).

The sub-fractions after gel column separation were finally purified by preparative HPLC. C18 reversed-phase column (250 mm×10 mm, 5 μM) was employed, with gradient elution by methanol-water or acetonitrile-water system (methanol: water = 30:70 to 100:0, elution time 60 min). Detection wavelengths of 210 nm, 254 nm, 365 nm (multi-wavelength detection to avoid missing target metabolites) were used (Feng et al., 2020). Flow rate of 3.0 mL/min, and column temperature at 30 °C were set up. The sub-fractions were dissolved in methanol, filtered through a 0.45 μM organic phase filter membrane, and injected into preparative HPLC. Single pure peaks were collected according to retention time, concentrated under reduced pressure, and freeze-dried (−50 °C, vacuum degree 0.09 MPa) to obtain monomeric metabolites as white powder or crystals (Sundarraj et al., 2018).

Modern spectroscopic techniques combined with physicochemical property analysis were used for structural elucidation of purified monomeric metabolites. Infrared spectroscopy (IR) and nuclear magnetic resonance spectroscopy (NMR) (^1^H-NMR (400MHz, CDCl_3_ or DMSO-d_6_ as solvent) and ^13^C-NMR (100MHz, same solvent as above) were employed. For some metabolites, two-dimensional nuclear magnetic resonance spectra such as DEPT, HSQC, HMBC, and ^1^H-^1^H COSY were supplemented to clarify the spatial connection between atoms (Zou et al., 2023). Electrospray ionization (ESI-MS) or electron impact ionization (EI-MS) was used to determine the molecular weight, molecular ion peak, and fragment ion peak of metabolites. Combined with NMR data, the molecular formula and structural fragments were deduced. Finally, the accurate structure of metabolites (Supplementary Figure S1) was confirmed by comparison with standard reference data or literature review.

### 2.3 Cell culture and assay

LX-2 (CL-0560), a human hepatic stellate cell line (batch number 240607032301), was purchased from Wuhan Promesa Life Science Technology Co., Ltd. (Lei et al., 2022). 56.2 mL fetal bovine serum (FBS) and 5.62 mL double antibiotics (penicillin, streptomycin) were added to 500 mL of DMEM medium. The aliquots in mL were stored at 4 °C in the refrigerator for use. PBS (pH = 7.35) containing 0.24 g potassium dihydrogen phosphate, 1.44 g potassium dihydrogen phosphate monohydrate, 0.2 g potassium chloride, and 8 g sodium chloride in 1000 mL of double-distilled water was sterilized and stored at 4 °C for future use. 50 μg of TGF-β1 protein powder was dissolved in 500 μL of sterile deionized water to prepare a 100 μg/mL TGF-β1 solution (Cao et al., 2021).

LX-2 cells were seeded at a concentration of 1×10^4^/mL in 96-well plates. After 4 h of culture, the cells were observed to adhere to the plate, and the supernatant was discarded. The cells were rinsed by PBS and introduced to the culture medium containing TGF-β1 (5, 10, 20, 40 ng/mL) for culturing at 24, 48, 72, 96, 120, 144, and 168 h. The absorbance of the cell culture was measured using the CCK8 kit (Solarbio, Beijing, China), with 3 replicates. The proliferation rate of LX-2 cells induced by each concentration of TGF-β1 was determined. The optimal concentration of TGF-β1 required for the assay model was selected based on the significant increase in proliferation rate (Lei et al., 2022).

The cell concentration was adjusted to 1×10^5^/mL and seeded in culture flasks. The cells were divided into two groups: normal blank group (vehicle control; solvent group), and model group (treated with 10 ng/mL TGF-β1). The cells were placed in the incubator for culturing. After normal cell recovery and passage, the cells were observed under a microscope for cell adhesion and growth. The cells were used, when LX-2 cells changed from small spindle-shaped to spindle-shaped (Tsuchida and Friedman, 2017).

Cell viability was determined by CCK8 method. The cell suspension was added to the 96-well plate (100 μL per well). The plate was placed in the incubator for pre-culture (37 °C, 5% CO_2_). 10 μL of CCK-8 solution were introduced to each well in a plate, and the plate was placed in the incubator for 3 h. Cell culture absorbance at 450 nm was measured using a miciroplate reader. Cell viability was calculated according to the following formula (Mosmann, 1983):

$$\text{Cell viability }\left(\%\right)=\left[A\;\left(\text{with drug}\right)‐A\;\left(\text{blank}\right)\right]/\left[A\;\left(0\text{ drug addition}\right)‐A\;\left(\text{black}\right)\right]$$

LX-2 cells were seeded at a concentration of 1×10^5^ cells/mL in 96-well plates. After 4 h of culture, when the cells adhered to the plate, the supernatant was discarded.

The cells were rinsed by PBS and introduced to the culture medium containing anti-liver fibrosis drug containing Chinese cordyceps (复方鳖甲软肝片). The concentrations of the positive group were set at 5 ng/mL, 10 ng/mL, 25 ng/mL, 75 ng/mL, and 100 ng/mL. The absorbance of the cell culture was measured using the CCK8 kit, with 6 replicates.

The inhibition rate was determined according to the following formula (Mosmann, 1983):

$$\text{Inhibition ratio }\left(\%\right)=\left[1‐\frac{\text{OD}450\;\left(\text{experimental}\;\text{group}\right)}{\text{OD}450\;\left(\text{blank}\;\text{group}\right)}\right]\times 100\%$$

The cells were inoculated at a concentration of 2.5×10^4^/mL in a 96-well plate, with 200 μL of DMEM complete culture medium per well. To promote more stable cell growth, the culture medium was removed after 24 h. The experiment was divided into the normal blank group and the TGF-β1-induced model group. The cells were cultured in the incubator for 120 h, and then all the culture medium of the model group was removed. 10 μM cerevisterol and 75 ng/mL anti-liver fibrosis drug (复方鳖甲软肝片), containing *Fructus forsythia* (16%), *Radix isatidis* (16%), *Carapax trionycis* (14%), *Radix codonopsis* (10%), *Radix paeoniae rubra* (10%), *Radix astragali* (10%), *Radix angelicae sinensis* (6%), *Radix notoginseng* (6%), *Placenta hominis* (5%), *Ophiocordyceps sinensis* (3%), and *Rhizoma curcumae* (3%) (Chen et al., 2000) (positive group) were added, and the cells were cultured for 48 h. The absorbance was measure by the CCK8 kit. The experiment was repeated 6 times, and the inhibition ratio was calculated (Mosmann, 1983):

$$\text{Inhibition ratio }\left(\%\right)=\frac{1‐\left[\text{OD}450\;\left(\text{experimental}\;\text{group}\right)‐\text{OD}450\;\left(\text{blank}\;\text{group}\right)\right]}{\text{OD}450\;\left(\text{model}\;\text{set}\right)‐\text{OD}450\;\left(\text{blank}\;\text{group}\right)}\times 100\%$$

### 2.4 qRT- PCR

RNA was extracted from the cells by Trizol reagent according to the manufacturing instruction. The qRT-PCR primers (Smad3-F: ACG ATG AGA CAG AGT TGC GAC, Smad3-R: ATC CAC GGC AGC AAT TCT CCC; Smad4-F: CAT CCC CAT GCC GCC GTT CA, Smad4-R: CTG CAG CTC TCC GGT CAG GA; Collagen I-F: CTG GCG GTT CAG GTC CAA T, Collagen I-R: TTC CAG GCA ATC CAC GAG C; β-actin-F:TGG CAC CCA GCA CAA TGA A, β-actin-R: CTA AGT CAT AGT CCG CCG AGA AGC A) (Xu et al., 2025) were synthesized by Shengong Biotechnology Engineering Co., Ltd. (Shanghai, China). The ReverTra Ace qPCR RT Kit (Guangzhou Xinkailai Biotechnology Co., Ltd.) was used. The reaction conditions were as follows: 37 °C for 2 min to remove genomic DNA contamination; 55 °C for 15 min; 85 °C for 5 min to terminate the reaction. The obtained products were quickly placed on ice or immediately stored at −20 °C for subsequent experiments. The β-actin gene was used as the internal reference for normalization of the target genes. The relative mRNA expression of each group was calculated using the 2^−ΔΔCT^ method (Lei et al., 2022).

### 2.5 Western blot analysis

Total protein in the cells was extracted using RIPA lysis buffer containing proteinase and phosphatase inhibitor (Beyotime, Shanghai, China). Total protein concentration was determined using a BCA protein assay kit (JL-T0336, Jonlnbio, Shanghai, China). Proteins (30–50 μg) were denatured at 100 °C for 5 min, and then separated by 8% or 10% sodium dodecyl sulfate–polyacrylamide gel electrophoresis. The proteins were electrotransferred onto polyvinylidene fluoride (PVDF) membranes (Qiao et al., 2023). The membranes were blocked in 5% BSA at room temperature for 60 min, and then incubated with primary antibody (Guangzhou Tianya Biotechnology) overnight at 4 °C. The membranes were incubated in the dark for 1 h at room temperature with fluorescence-labeled secondary antibody (Guangzhou Tianya Biotechnology). The PVDF membranes were scanned using the Odyssey 2.1 software of Odyssey infrared scanner (LI-COR Biosciences, Lincoln, NE, United States). After scanning, target protein bands were cut out according to the molecular weight of target protein without any edit. The greyscale values relative to β-actin of the target proteins were analyzed using ImageJ software (Eremeev et al., 2021).

### 2.6 Data processing

The data were analyzed using SPSS 22.0 statistical software (Chicago, United States). The measurement data were expressed as the mean ± SD, and multivariate analysis of variance was conducted for the data statistics. GraphPad Prism 8.0.2 software was used for plotting (Qiao et al., 2023). If the variances were homogeneous, pairwise comparisons between multiple groups were performed by the oneway analysis of variance, followed by the least significant difference test. p < 0.05 was considered statistically significant; if the variances were not homogeneous, the Tamhane T2 test was used.

## 3 Results

### 3.1 Structure identification

**FIGURE 1:**
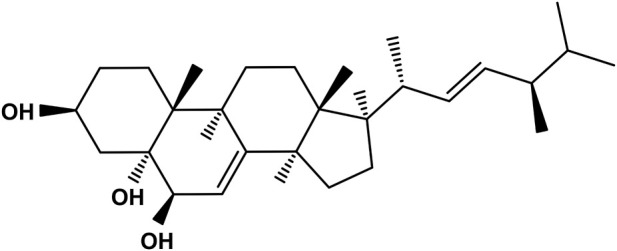
Chemical structure of cerevisterol.

Metabolite: white powder, C_28_H_46_O_3_, ^1^H NMR (600 MHz, Chloroform-d) δ 5.35 (dt, J = 4.9, 2.2 Hz, 1H), 5.29 – 5.12 (m, 2H), 4.08 (tt, J = 10.7, 4.7 Hz, 1H), 3.62 (s, 1H), 3.49 (s, 2H), 2.18 – 2.10 (m, 1H), 2.05 (ddt, J = 15.2, 13.1, 5.1 Hz, 2H), 1.94 (ddd, J = 27.5, 12.1, 6.6 Hz, 2H), 1.84 (s, 2H), 1.82 – 1.67 (m, 2H), 1.63 – 1.51 (m, 10H), 1.46 (dddd, J = 23.5, 21.0, 10.7, 5.3 Hz, 4H), 1.39 – 1.19 (m, 4H), 1.09 (s, 2H), 1.03 (d, J = 6.6 Hz, 2H), 0.92 (d, J = 6.8 Hz, 2H), 0.83 (dd, J = 9.2, 6.8 Hz, 4H), 0.60 (s, 2H); 13C NMR (126 MHz, CDCl_3_) δ 144.01, 135.36, 132.17, 117.52, 77.25, 77.00, 76.75, 73.66, 67.72, 55.97, 54.74, 43.75, 43.46, 42.80, 40.39, 39.45, 39.20, 37.13, 33.06, 32.95, 30.84, 29.69, 27.89, 22.87, 22.03, 21.10, 19.93, 19.63, 18.83, 17.58, 12.32 (Figure 1). The above data are basically consistent with literature reports (Gao et al., 2007). The metabolite was identified as cerevisterol.

### 3.2 Screening of TGF-β1 concentration and time for LX-2 cell induction

LX-2 cells were cultured in 96-well plates at 37 °C. After adherent growth, the cells were treated with 5, 10, 20, 40 ng/mL of TGF-β1 for 24 h, 48 h, 72 h, 96 h, and 120 h, respectively. Each concentration showed a promoting effect on cell proliferation as shown in Supplementary Figure S2. With the increase of time, the cell number increased and the survival rate rose. However, at 120 h, the cell viability decreased, possibly because the nutrients in the culture medium were exhausted due to the long incubation time. Compared with other concentrations of TGF-β1, the OD450 of cells induced by 10 ng/mL TGF-β1 increased significantly at 72 h, with the proliferation rate reaching up to 66.87% (*F* = 85.79; *df* = 3; *p* < 0.01). Finally, 10 ng/mL TGF-β1 was selected to treat LX-2 cells.

After resuscitation and passage of LX-2 cells, the cells were observed under a microscope. The cell morphology was epithelial-like, polygonal, adherent, transparent and round in shape, closely arranged, and of uniform size. As shown in Supplementary Figure S3, after 72 h of induction by 10 ng/mL TGF-β1, it was clearly observed that the cells gradually changed from their original round shape to oval and short spindle shapes, and the cell morphology changed. Therefore, 72 h was selected as the optimal time for establishing the TGF-β1-induced liver fibrosis model in LX-2 cells.

### 3.3 The inhibitory effect of cerevisterol at different concentrations on LX-2 cells

**FIGURE 2:**
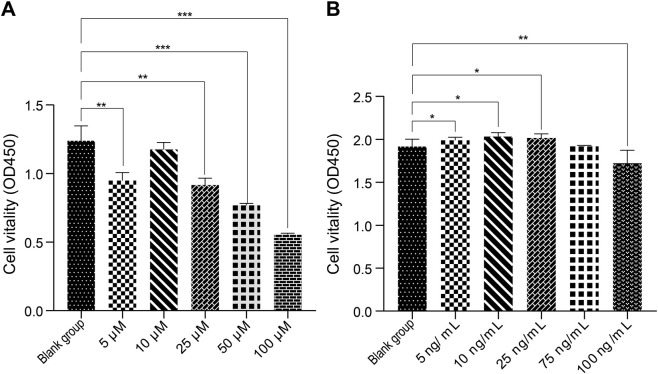
Effect of different concentrations of cerevisterol **(A)** and the anti-liver fibrosis drug containing Chinese cordyceps **(B)** on the proliferation of LX-2 cells. Statistical significance was defined as *p < 0.05, **p < 0.01, ***p < 0.001.

After 24 h, different concentrations of cerevisterol had a significant effect on the growth of LX-2 cells. Compared with the control (Figure 2A), the cerevisterol at concentrations of 5, 25, 50, and 100 μM had inhibitory effect on the cell viability, with the lowest inhibitory effect at 10 μM. The anti-liver fibrosis drug containing Chinese cordyceps (positive group) had a relatively low effect on cell growth (Figure 2B). There was no significant difference in cell viability (OD450) between the drug 75 ng/mL and the control group, so the drug 75 ng/mL was selected as the optimal growth concentration for LX-2 cells.

### 3.4 The effect of cerevisterol on the proliferation of TGF-β1-induced LX-2 cells

**FIGURE 3:**
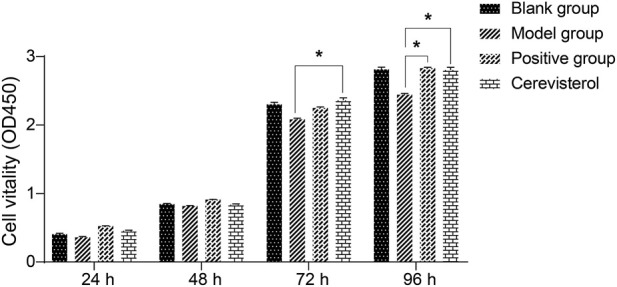
Effect of cerevisterol (10 μM) on the proliferation of the TGF-β1-induced LX-2 cells. Cell viability (absorbance at 450 nm) was assessed following a 4-h incubation. Cells were assigned to four experimental groups: (i) blank control (no TGF-β1 stimulation), (ii) model group (stimulated with 10 ng/mL TGF-β1), (iii) positive group (TGF-β1-stimulated LX-2 cells treated with 75 ng/mL of the anti-liver fibrosis drug containing Chinese cordyceps), and (iv) cerevisterol group (TGF-β1-stimulated LX-2 cells treated with 10 μM cerevisterol). * Significant difference at p < 0.05.

After the cells were cultured in 96-well plates for 4 h, they were divided into blank group, model group (TGF-β1: 10 ng/mL), positive group (the anti-liver fibrosis drug containing Chinese cordyceps: 75 ng/mL), and cerevisterol group (10 μM). In the positive group and the cerevisterol group, 10 ng/mL of TGF-β1 inducer was also added. The results were detected by the microplate reader. The cell growth rate at 72 h was the highest compared to those at 24 h and 48 h. Moreover, at 72 h, there was a significant difference between the cerevisterol group and the blank group (*F* = 87.5; *df* = 3; *p* < 0.05), and at 96 h, there were significant differences between the positive group, the cerevisterol group and the control group (*P < 0.05) (Figure 3).

### 3.5 The effect of cerevisterol on the transcriptional levels of related genes of TGF-β1-induced LX-2 cells

**FIGURE 4:**
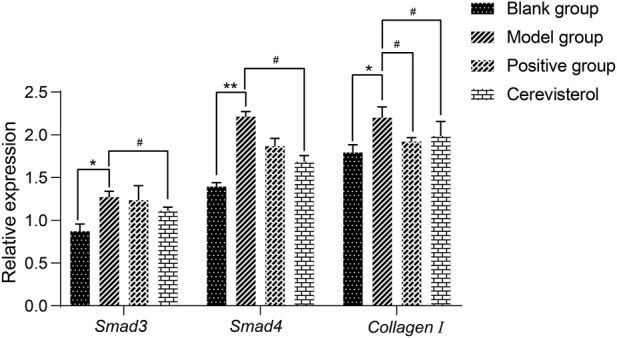
Effects of cerevisterol and the anti-liver fibrosis drug on the mRNA expression levels of *Smad3*, *Smad4* and *Collagen I* genes in TGF-β1-induced LX-2 cells. The mRNA expression levels were quantified by qRT-PCR in the four experimental groups (the blank group, model group, positive group and cerevisterol group; please see Figure 3). *p < 0.05, **p < 0.01 versus blank group; #p < 0.05 versus model group.

LX-2 cells were cultured in petri dishes for 4 h at 37 °C. Then, the cells were treated with TGF-β1 at 10 ng/mL (as model group), TGF-β1 at 10 ng/mL and the anti-liver fibrosis drug containing Chinese cordyceps at 75 ng/mL (as positive group), and TGF-β1 at 10 ng/mL and cerevisterol at 10 μM (as cerevisterol group) for 72 h. Daily observation, recording and photography were conducted in a sterile condition, to avoid contamination. As shown in Figure 4, qRT-PCR results indicated that TGF-β1 could promote the expression of *Smad3*, *Smad4* and *Collagen I* in the cells. Compared with the blank group (without TGF-β1-induced), the relative mRNA expression levels of *Smad3* (*F* = 131.52; *df* = 5; *p* < 0.01), *Smad4* (*F* = 112.9; *df* = 5; *p* < 0.01) and *Collagen I* (*F* = 66.81; *df* = 5; *p* < 0.01) in the model group significantly increased. Compared with the model group, the relative mRNA expression levels of *Smad3* (*F* = 75.73; *df* = 5; *p* < 0.01), *Smad4* (*F* = 18.54; *df* = 5; *p* < 0.05) and Collagen I (*F* = 66.81; *df* = 5; *p* < 0.01) in the cerevisterol group significantly decreased, and the relative mRNA expression level of *Collagen I* in the positive group also significantly decreased (*F* = 66.81; *df* = 5; *p* < 0.01).

### 3.6 Western blot

**FIGURE 5:**
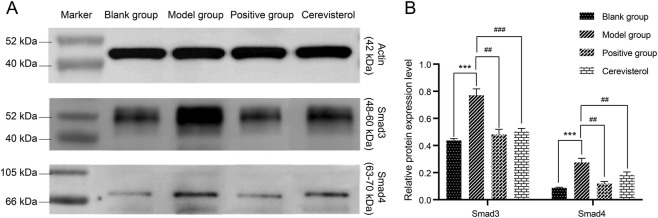
Relative expression of Smad3 and Smad4 proteins in TGF-β1-induced LX-2 cells treated by cerevisterol. The protein relative expression levels were quantified by Western blot in the four experimental groups (the blank group, model group, positive group and cerevisterol group; please see Figure 3). The protein expression level of Actin was used as internal reference, and band intensities were quantified using ImageJ in the chart **(A)** ***, compared with the blank group, significant difference at p < 0.001; ###, compared with the model group, significant difference at p< 0.001; ##, at p < 0.01 in the chart **(B)**.

As shown in Figure 5, compared with the blank group, the relative expression levels of Smad3 and Smad4 proteins in the model group significantly increased (*F* = 131.52; *df* = 5; *p* < 0.001). Compared with the model group, the relative expression levels of Smad3 and Smad4 proteins in the positive group and the cerevisterol group significantly decreased (*F* = 64.92; *df* = 5; *p* < 0.001), which also indicated that cerevisterol effectively reduced the expression levels of proteins in TGF-β1-induced LX-2 cells.

## 4 Discussion

Chinese cordyceps derived bioactive metabolites such as polysaccharides are used for liver protection (Cheng et al., 2014; Liu and Shen, 2003; Wu et al., 2017). However, exact molecules with better anti-inflammatory function from Chinese cordyceps or *O. sinensis* fungus needs to be explored. In this study, cerevisterol was first identified from *O. sinensis* fruiting bodies and used to evaluate its effect in the TGF-β1-induced fibrosis model. Cerevisterol at 10 μM and the positive control at 75 ng/mL significantly suppressed cell proliferation at 72 and 96 h, and downregulated the mRNA expression of Smad3, Smad4, and Collagen I in TGF-β1-induced LX-2 cells, which was confirmed by Western blot. These findings indicate that the cerevisterol from *O. sinensis* fruiting bodies holds potential as a therapeutic agent for liver fibrosis.

Although Chinese cordyceps or *O. sinensis* fungus exhibits remarkable hepatoprotective effects, especially anti-inflammatory and anti-fibrosis (Cheng et al., 2014; Liu and Shen, 2003; Peng et al., 2014a; Peng et al., 2014b; Wu et al., 2017; Fu et al., 2021; Peng et al., 2013), so far, only polysaccharides are regarded as the Chinese cordyceps-derived bioactive metabolites for hepatoprotective effects (Peng et al., 2013). Cerevisterol is identified from the Chinese cordyceps (Li et al., 2003; Long et al., 2021), *Myrothecium roridum* (Shen et al., 2010), *Viola diffusa* (Dai et al., 2015), *Cordyceps morakotii* (Wang et al., 2019), *Fusarium solani* (Alam et al., 2020), the fruiting bodies of the Thai wild edible mushroom *Astraeus asiaticus* (Maliwong et al., 2025), and *Poria cocos* (Liu et al., 2024; Wong et al., 2023). This metabolite is beneficial to anti-hepatitis B virus (Dai et al., 2015), anti-inflammatory action (Alam et al., 2020), anti-hyperuricemia (Huang et al., 2023), anti-asthma (Lin et al., 2024) and pustular acne by oxidative stress (Bowe et al., 2012). Cerevisterol sought from pharmacological databases of *P*. *cocos* were identified as a candidate metabolite for weight management, due to their high affinities, high drug-likeness and low toxicity profiles (Wong et al., 2023), indicating its effectiveness and clinical feasibility. However, no report on the ultilization of cerevisterol for liver fibrosis is found, although some cues are presented based on network pharmacology analysis and molecular docking (Zhang et al., 2022). The results in this study demonstrate that cerevisterol can be used for liver fibrosis, exerting comparable efficacy with the commercial prescription drug in the present market in China, but the cost for cerevisterol is much cheaper than that for the prescription drug.

In addition to the direct effects on hepatic stellate cells and signaling pathways, cerevisterol may also contribute to the anti-fibrotic process through their anti-inflammatory and antioxidant properties. Chronic inflammation and oxidative stress are often associated with liver injury and subsequent fibrosis (Arthur and Fibrogenesis, 2000). Some phytosterols have been reported to possess anti-inflammatory activities by suppressing the production of pro-inflammatory cytokines such as TNF - α and IL - 6 (similar to the effects of other natural metabolites with anti-fibrotic potential). By reducing inflammation, cerevisterol may alleviate the damage to hepatocytes and the subsequent activation of hepatic stellate cells. Their antioxidant properties may also help to counteract the oxidative stress-induced activation of fibrogenic pathways in the liver. Moreover, a pilot study on sub-micron dispersible free phytosterols demonstrated their efficacy in improving liver steatosis, which is often associated with the early stages of liver fibrosis development in non-alcoholic fatty liver disease (NAFLD) patients (Brañes et al., 2023). Given the structural similarities among phytosterols, it is reasonable to hypothesize that cerevisterol may also have a positive impact on NAFLD-related fibrosis.

Several aspects regarding the anti-fibrotic effects of cerevisterol require further investigation. The optimal dosage and treatment duration for achieving significant anti-fibrotic effects in humans remain to be determined. Additionally, the bio-availability of cerevisterol needs to be improved, as their low solubility may limit their delivery to the target tissues in the liver. Future research could explore novel drug delivery systems to enhance their efficacy. This study is only based on in-vitro only nature, by using TGF-β1-induced LX-2 cells with no animal validation. Further research should be done with animal models to fully validate the function of cerevisterol.

In conclusion, current evidence suggests that cerevisterol holds promise as a potential anti-hepatic fibrotic agent. Their multi-faceted mechanisms of action, including modulation of hepatic stellate cells activation, TGF-β1/Smad3 signalling, and anti-inflammatory/antioxidant effects, make them an interesting candidate for further pre-clinical and clinical studies. If its anti-fibrotic potential is fully validated, cerevisterol could offer a new therapeutic option for patients suffering from liver fibrosis.

## Data Availability

The original contributions presented in the study are included in the article/Supplementary Material, further inquiries can be directed to the corresponding authors.
