# Supplementary material for: Cerevisterol from Ophiocordyceps sinensis fruiting bodies against liver fibrosis
Source: Front Pharmacol. 2026 Jul 8;17:1825109. doi: 10.3389/fphar.2026.1825109 (PMC13388481; doi:10.3389/fphar.2026.1825109)
Supplement: Supplementary file 1 [file DataSheet2.zip › Raw data/Supplementary Materials/Figure S1.docx]

**A B**

**
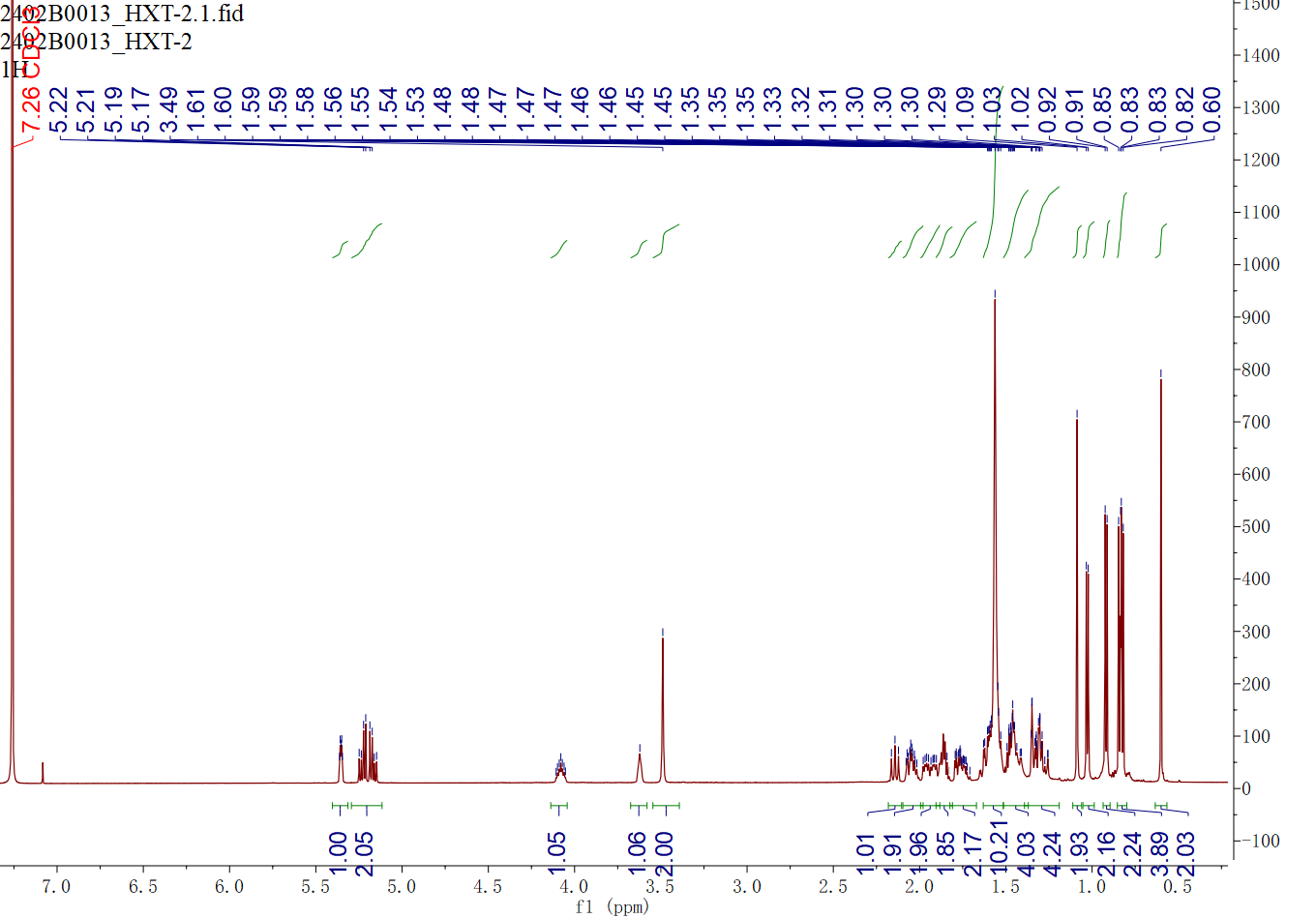

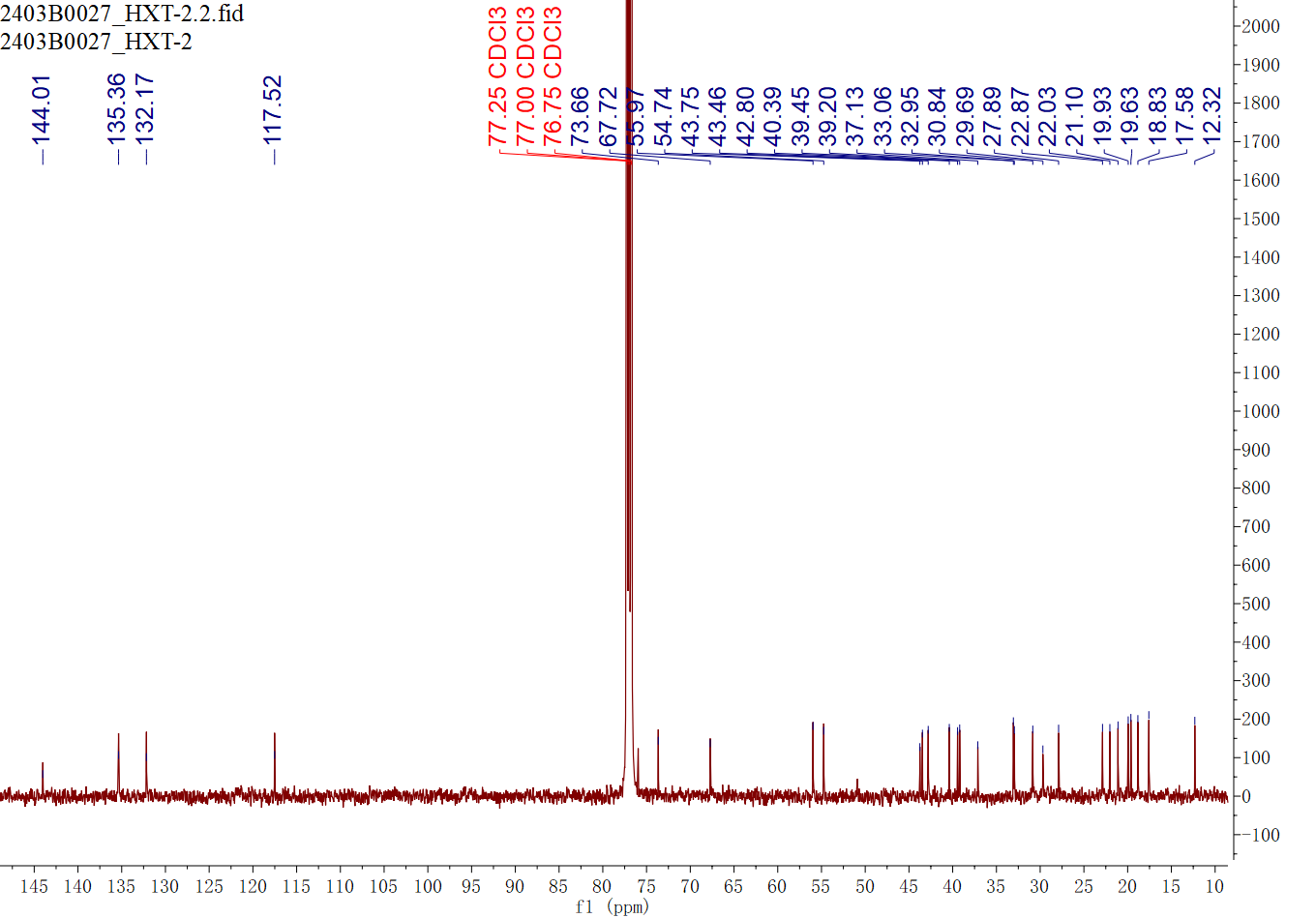
**

**C D**

**
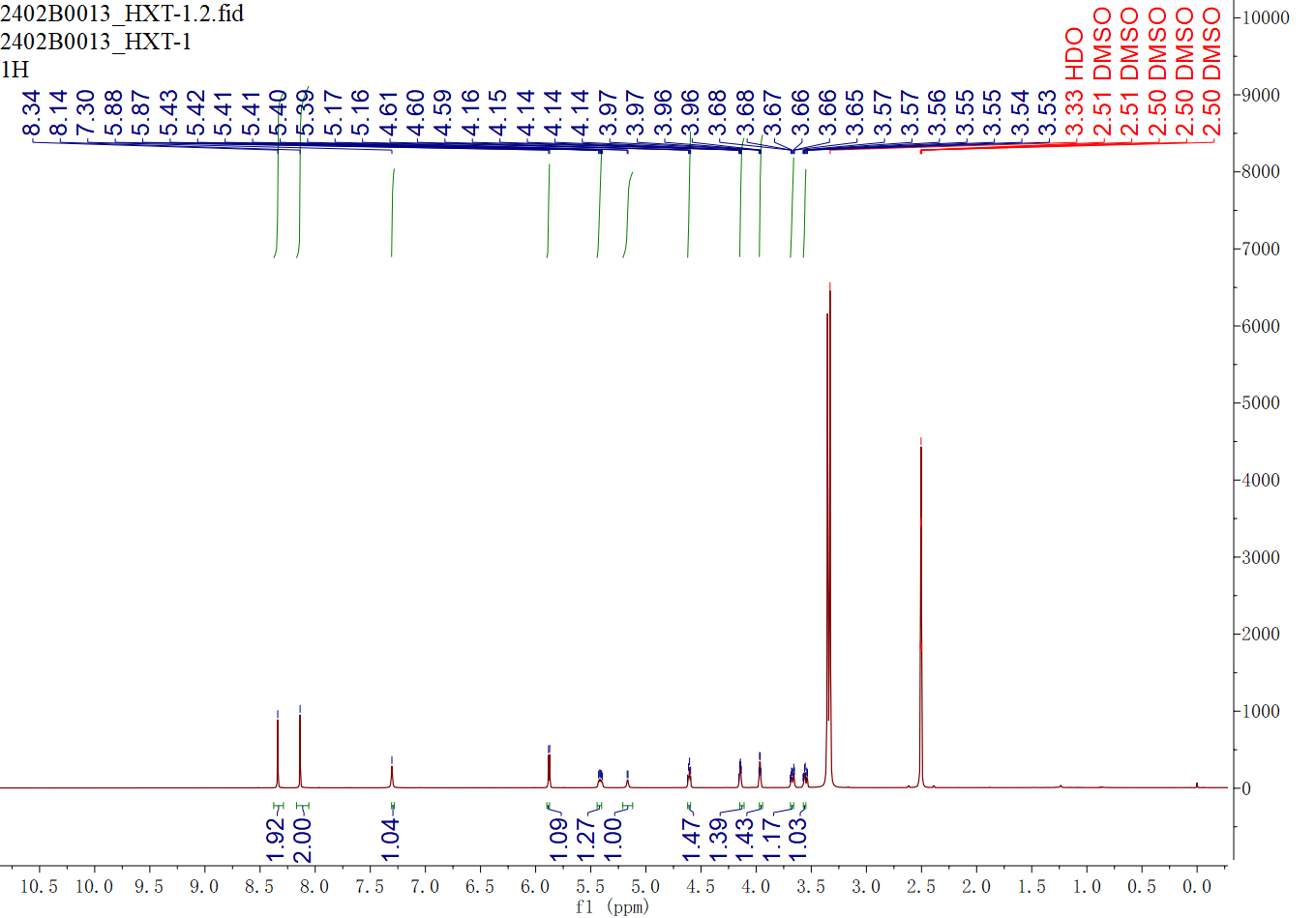

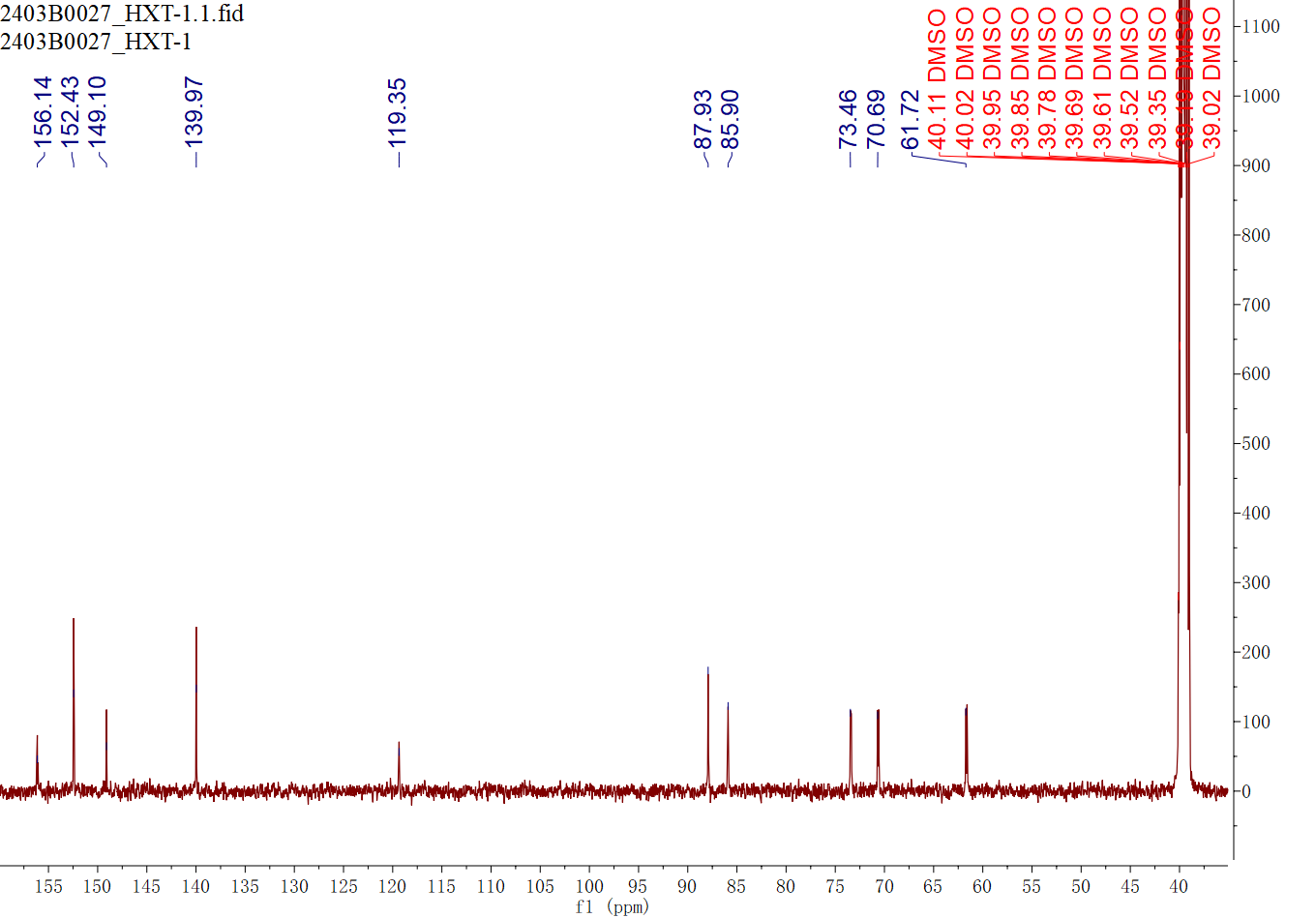
**

**E F**

**
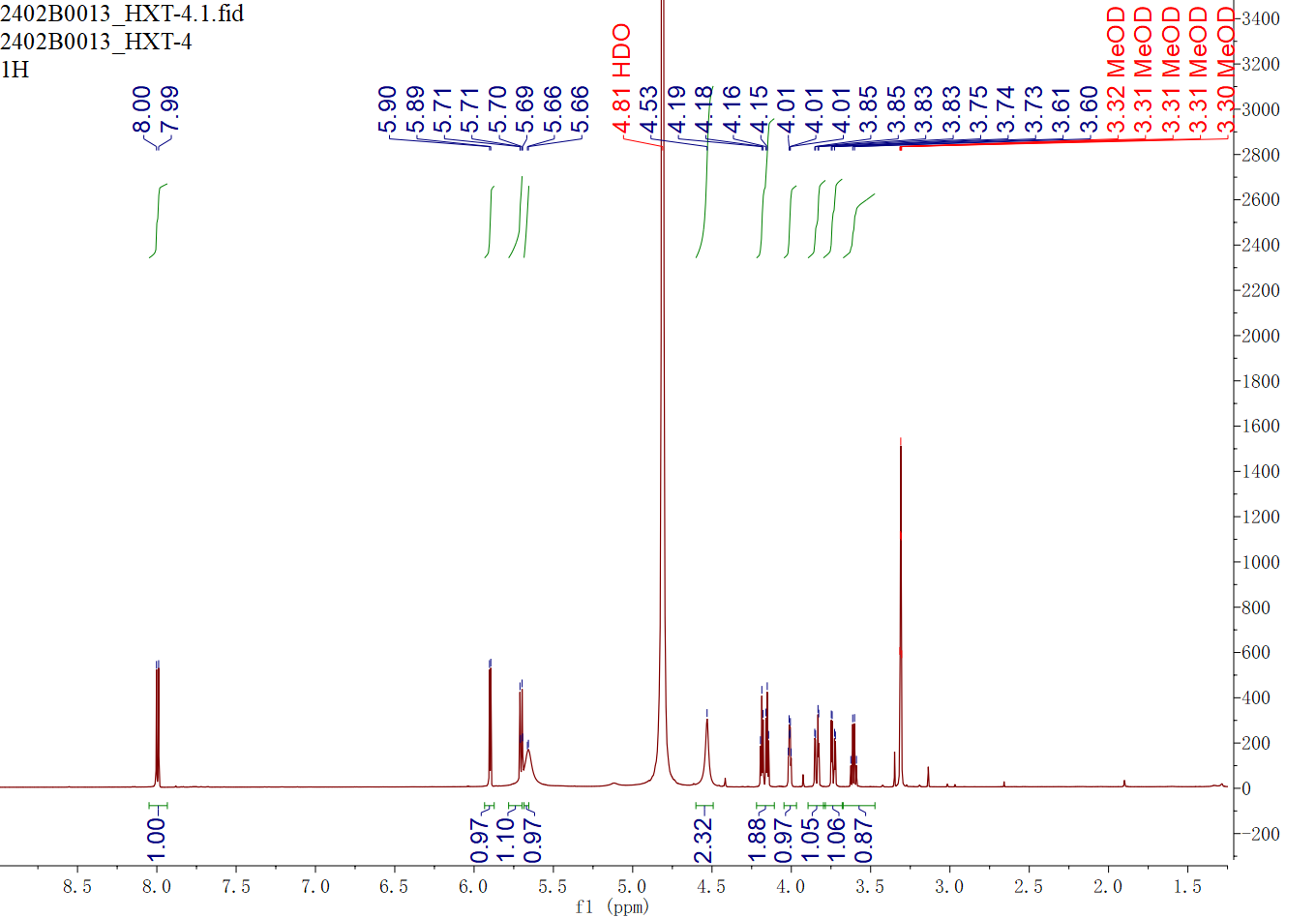

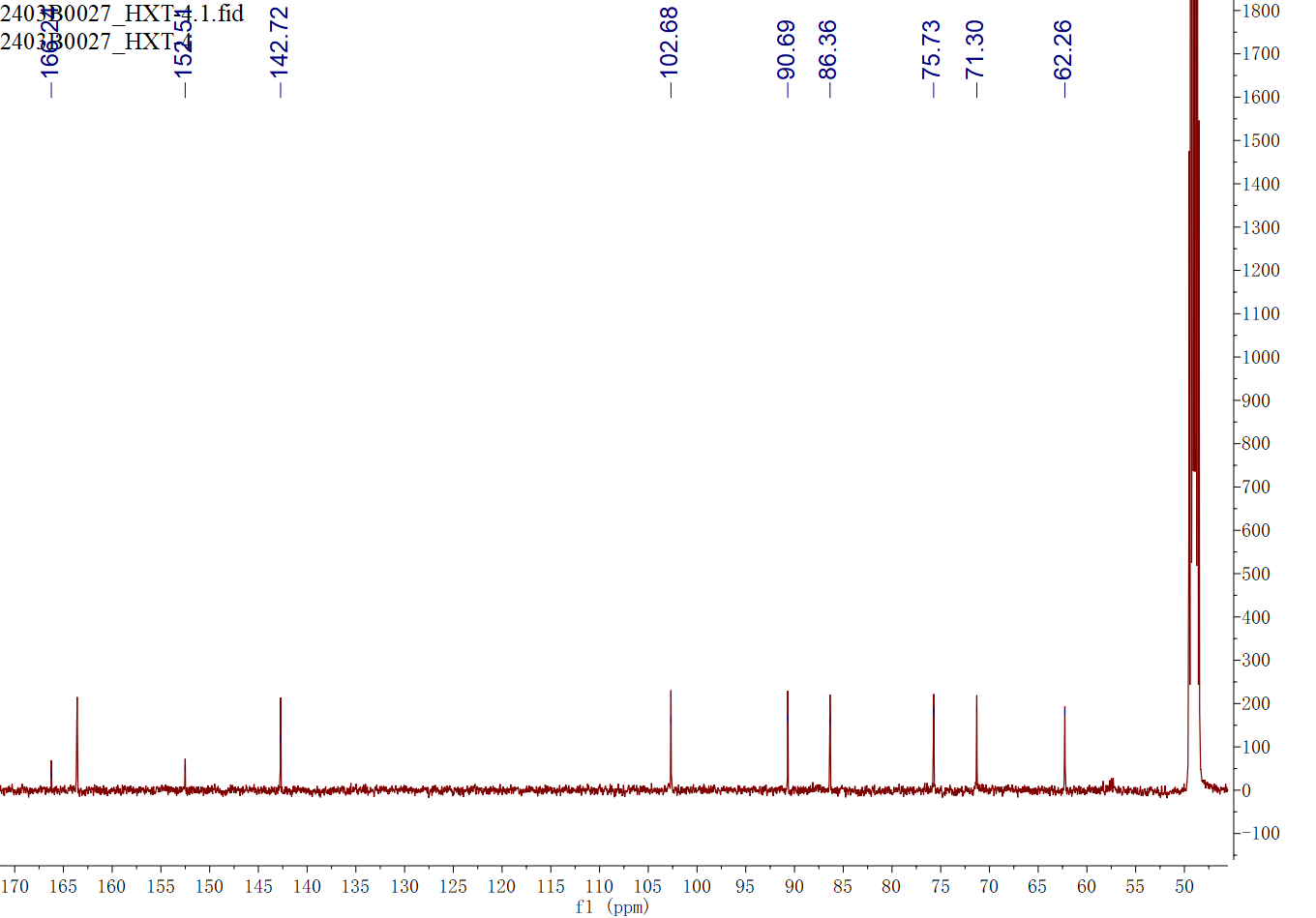
**

**G H**

**
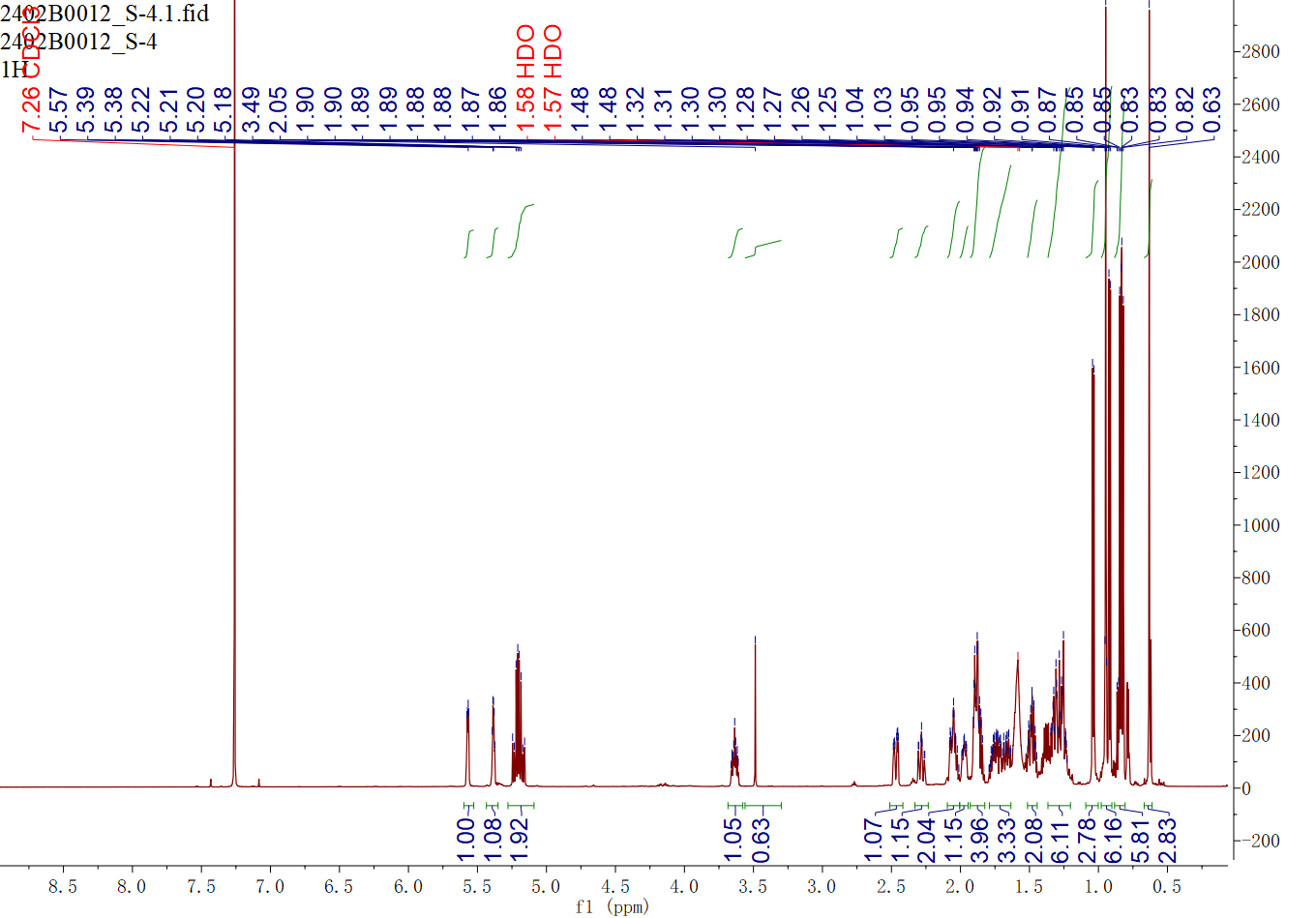

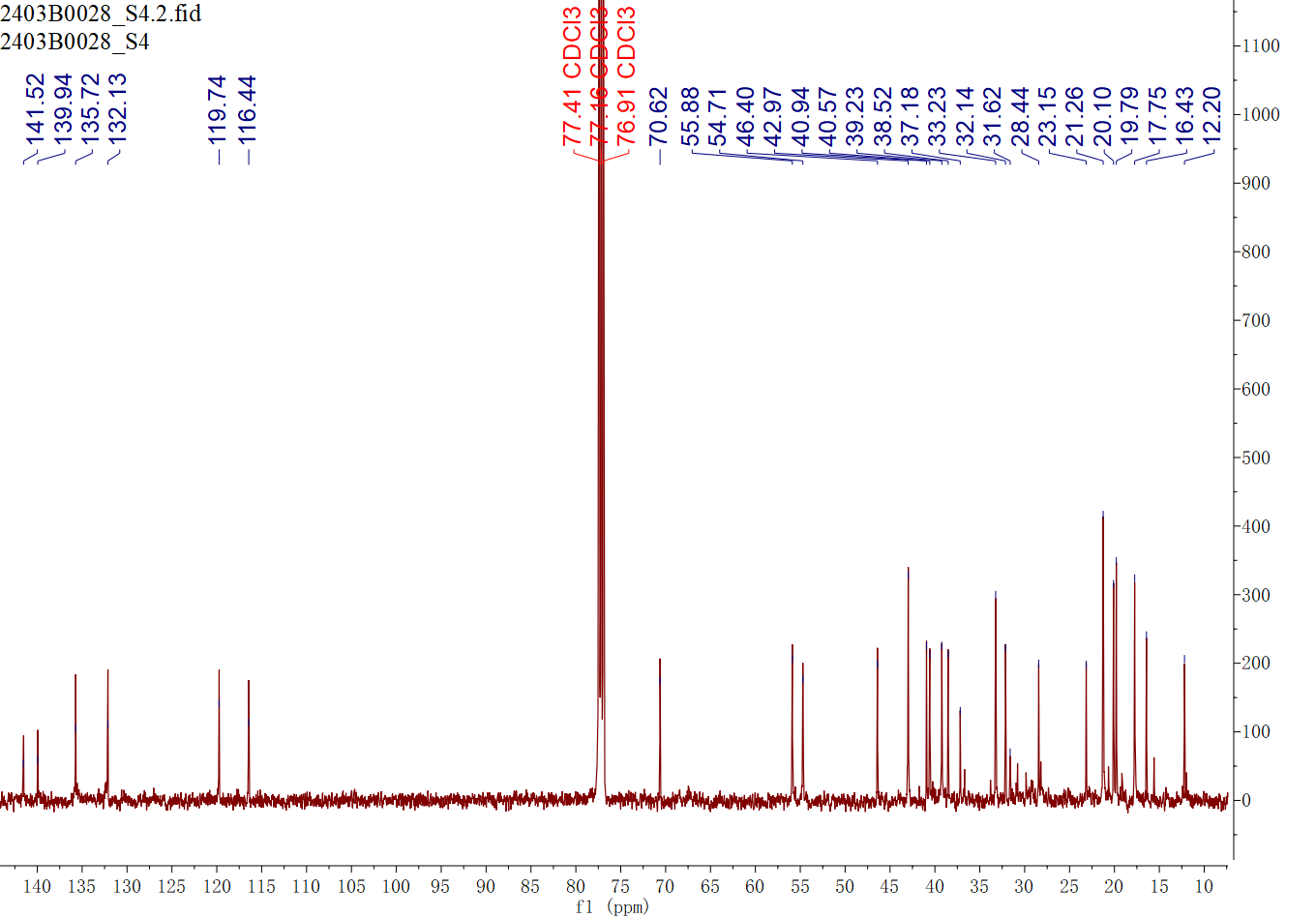
**

**I J**

**
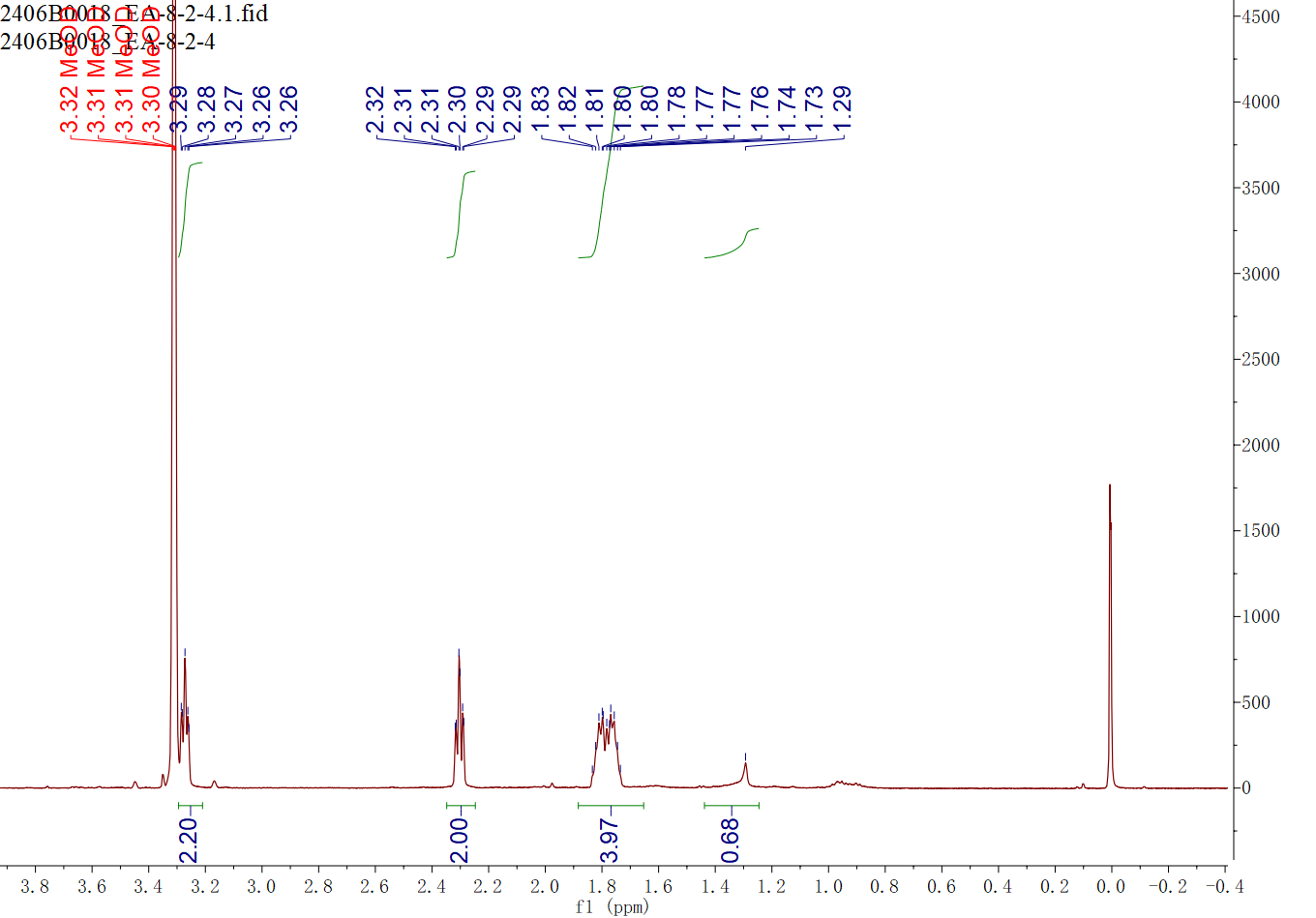

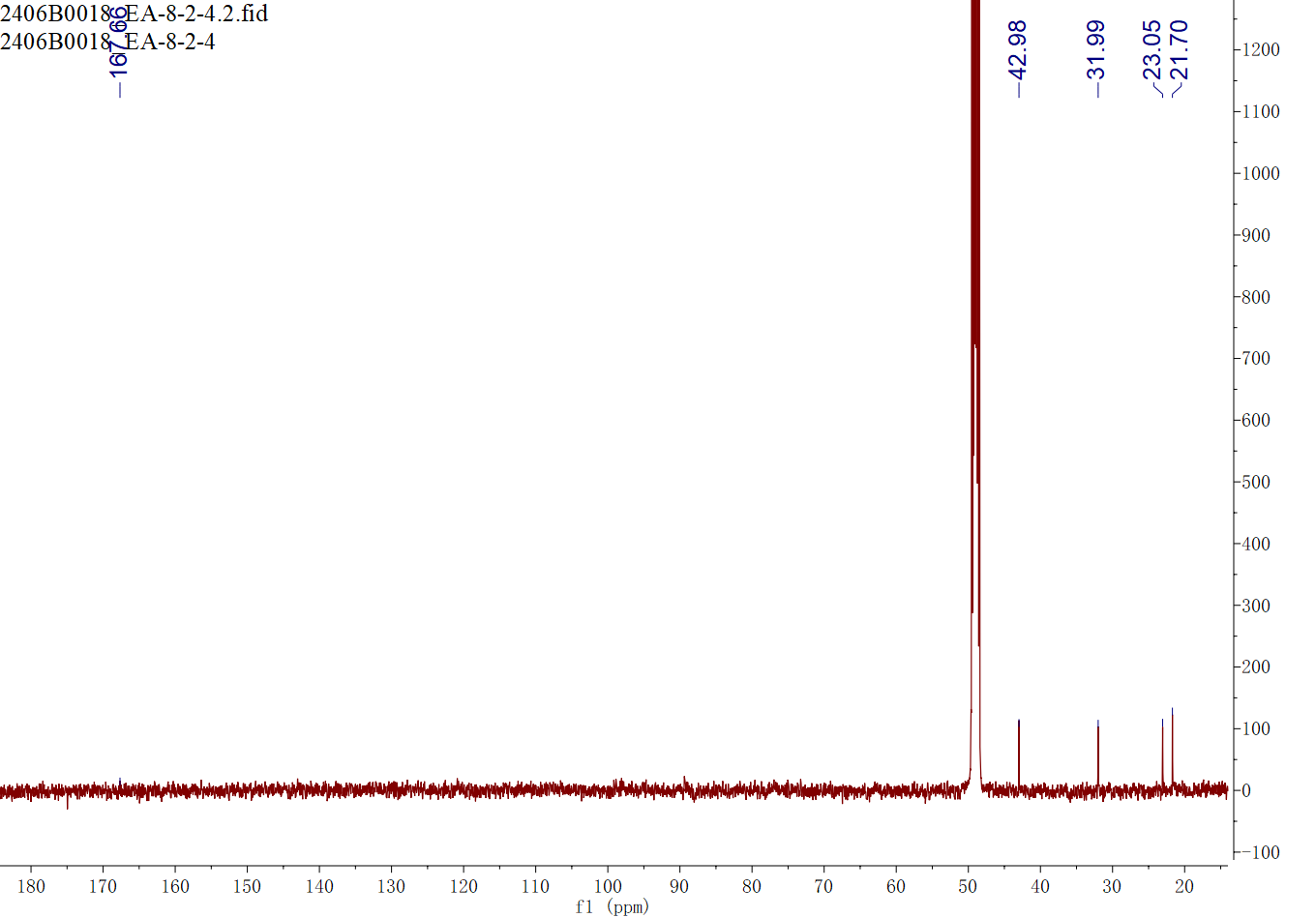
**

**K L**

**
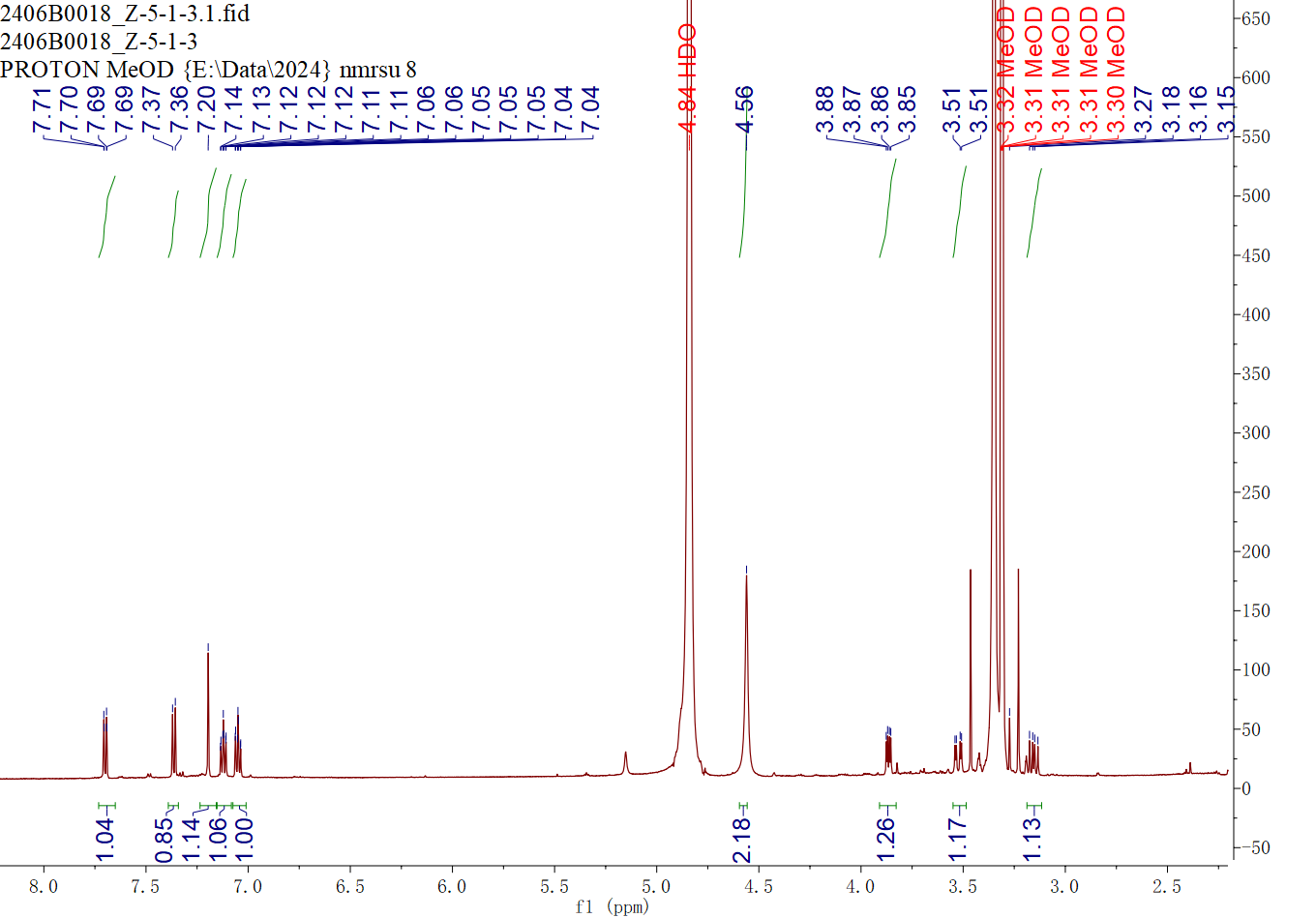

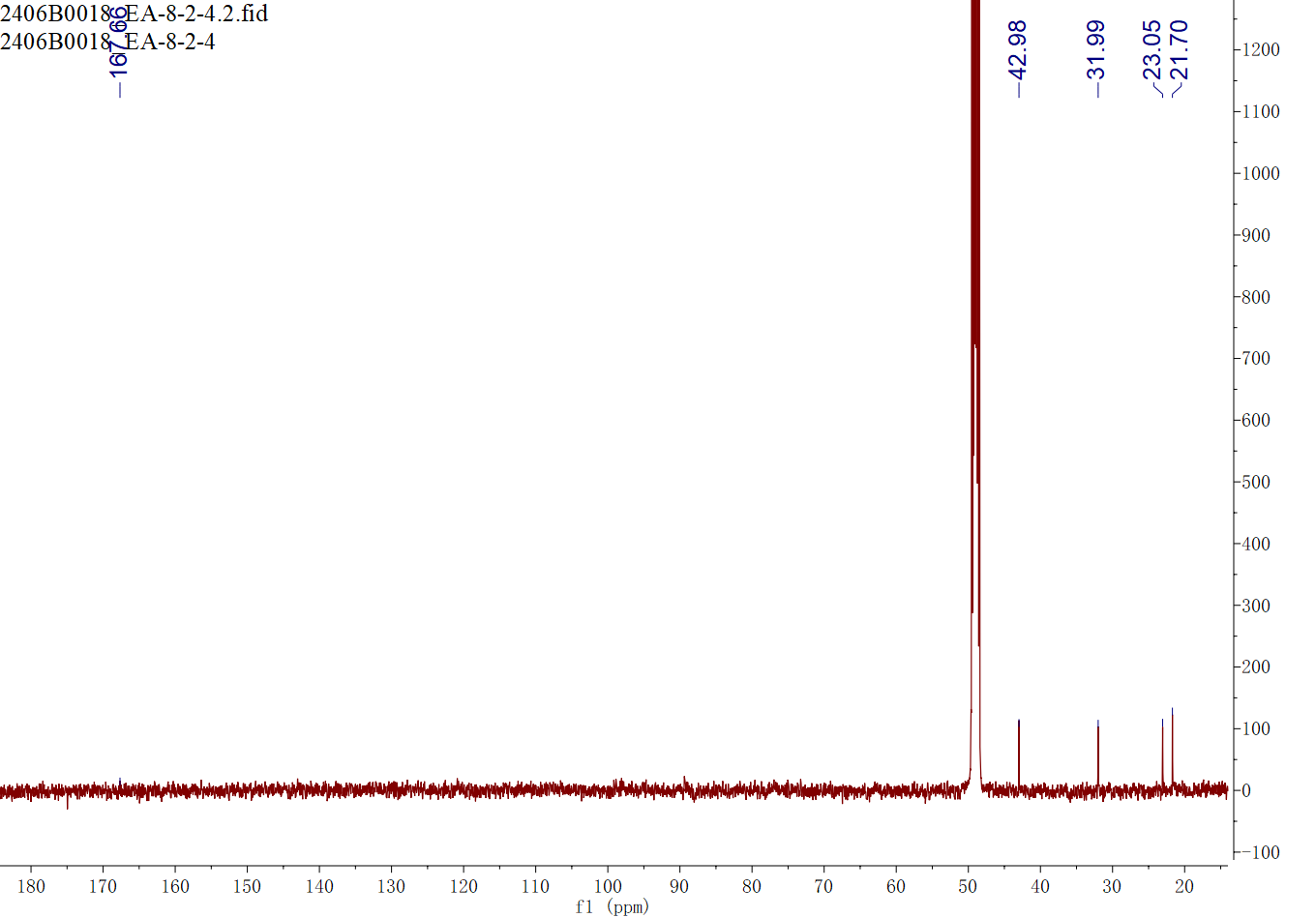
**

**M N**

**
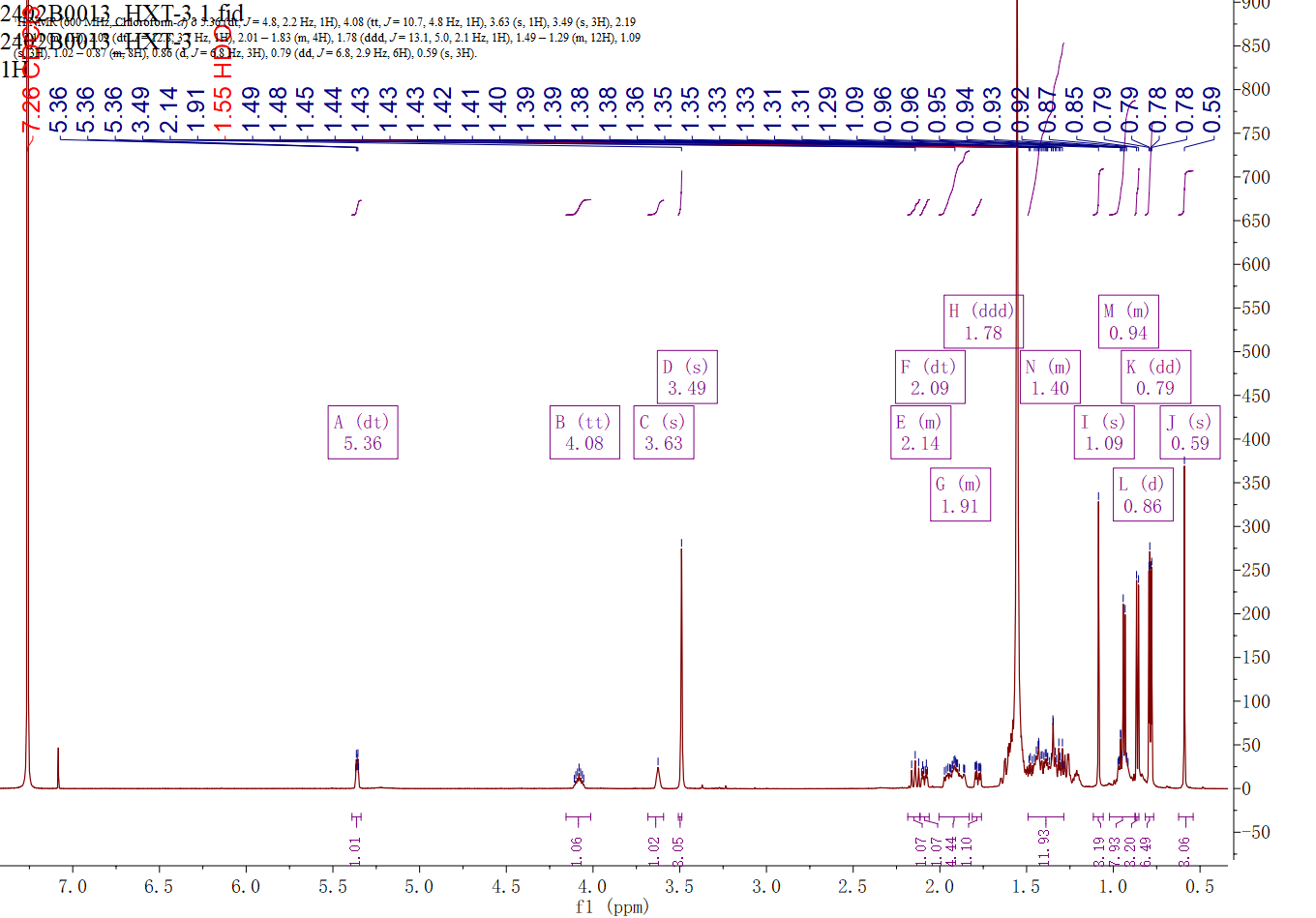

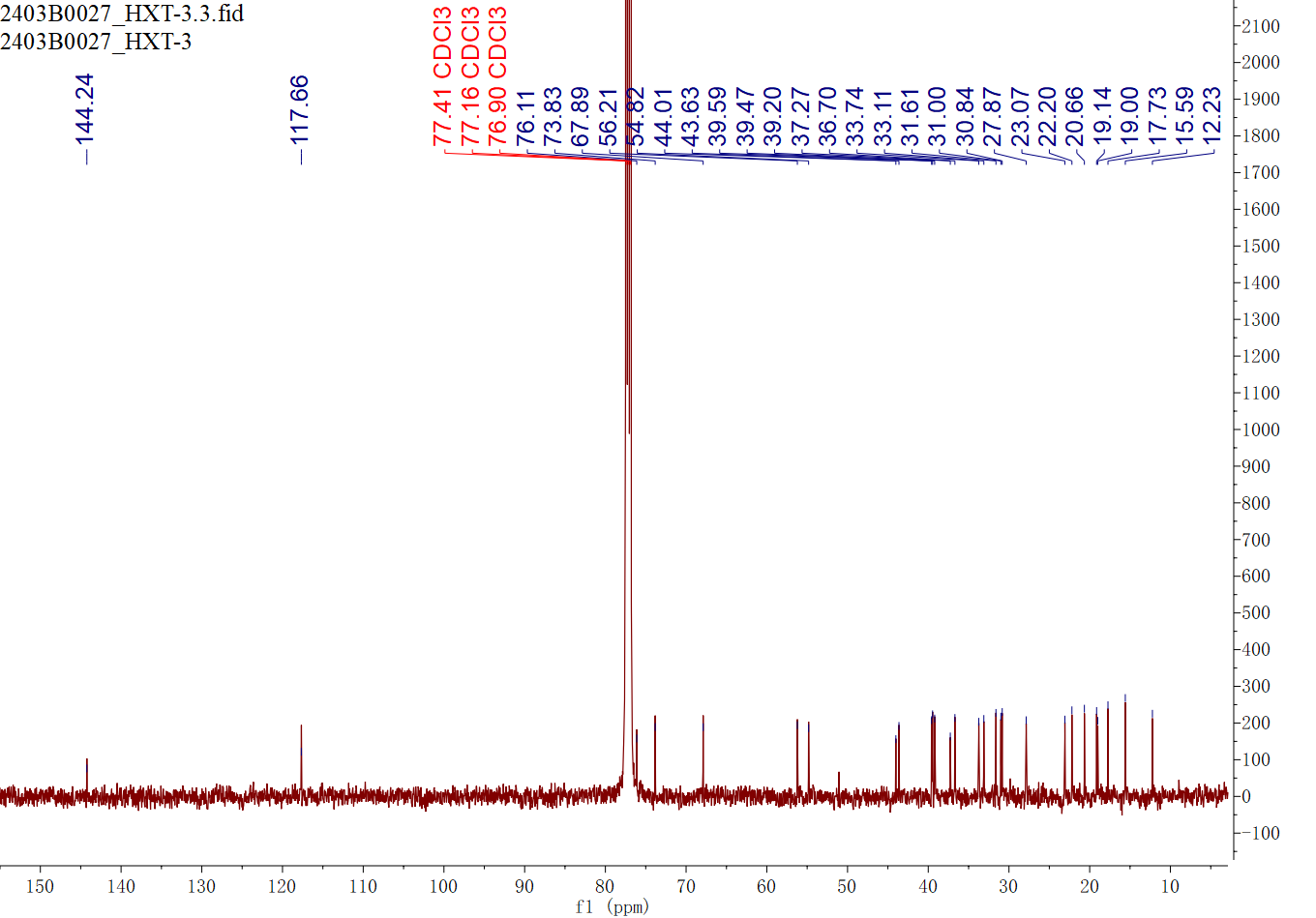
**

**
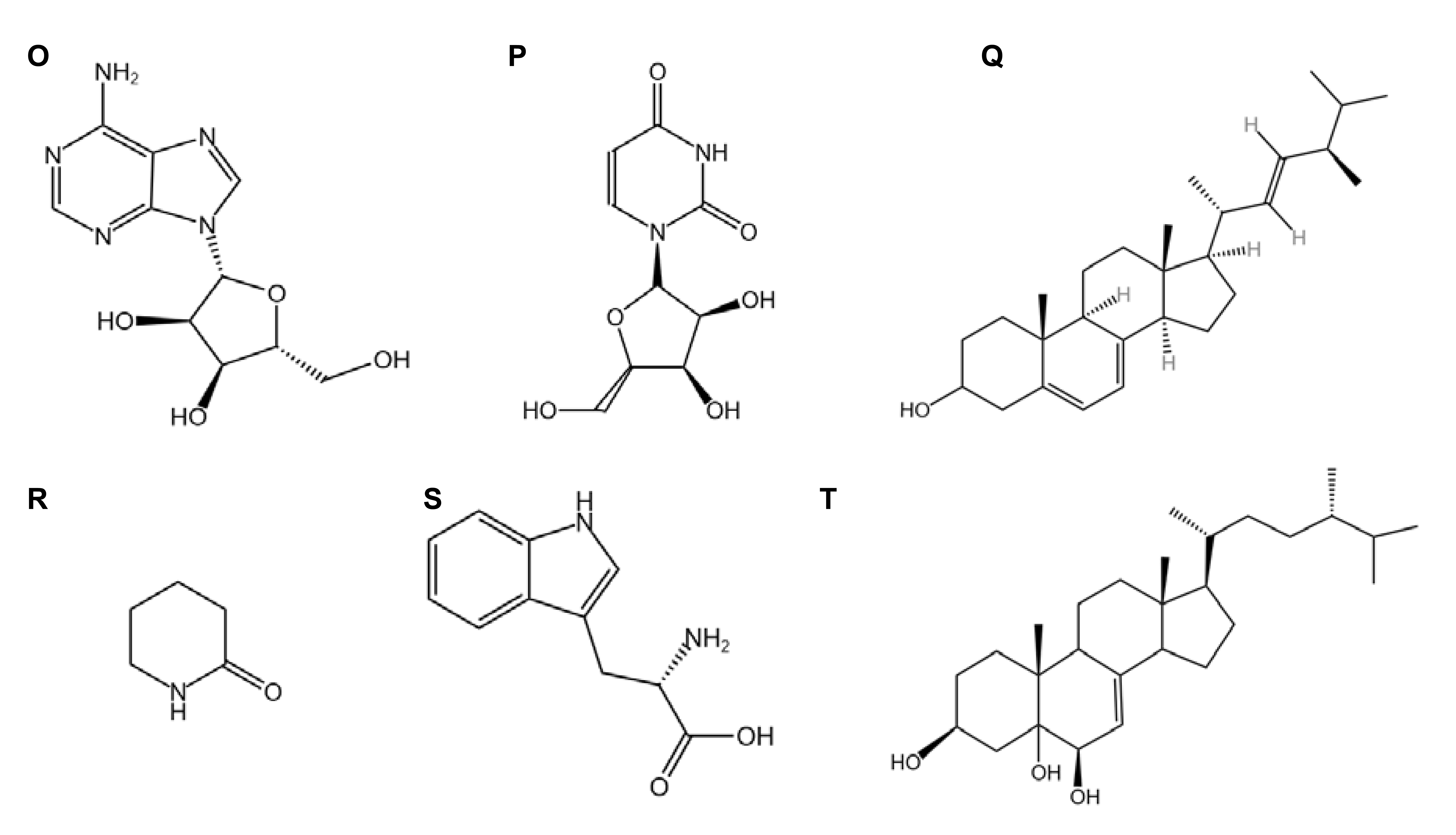
**

Figure S1. ^1^H NMR and ^13^C NMR spectra of the compounds extracted and obtained from *Ophiocordyceps sinensis* fruiting bodies. A and B: ^1^H NMR and ^13^C NMR spectra of cerevisterol; C and D: ^1^H NMR and ^13^C NMR spectra of adenosine; E and F: ^1^H NMR and ^13^C NMR spectra of uridine; G and H: ^1^H NMR and ^13^C NMR spectra of ergosterol; I and J: ^1^H NMR and ^13^C NMR spectra of 2-piperidone; K and L: ^1^H NMR and ^13^C NMR spectra of L-tryptophan; M and N: ^1^H NMR and ^13^C NMR spectra of (24S)-ergost-7(8)-en-3β,5α,6β-triol; O, P, Q, R, S, and T correspond to the chemical structures of adenosine, uridine, ergosterol, 2-piperidone, L-tryptophan, and (24S)-ergost-7(8)-en-3β,5α,6β-triol, respectively.
