# Supplementary material for: Cerevisterol from Ophiocordyceps sinensis fruiting bodies against liver fibrosis
Source: Front Pharmacol. 2026 Jul 8;17:1825109. doi: 10.3389/fphar.2026.1825109 (PMC13388481; doi:10.3389/fphar.2026.1825109)
Supplement: Supplementary file 1 [file DataSheet2.zip › Raw data/Supplementary Materials/Figure S2.docx]

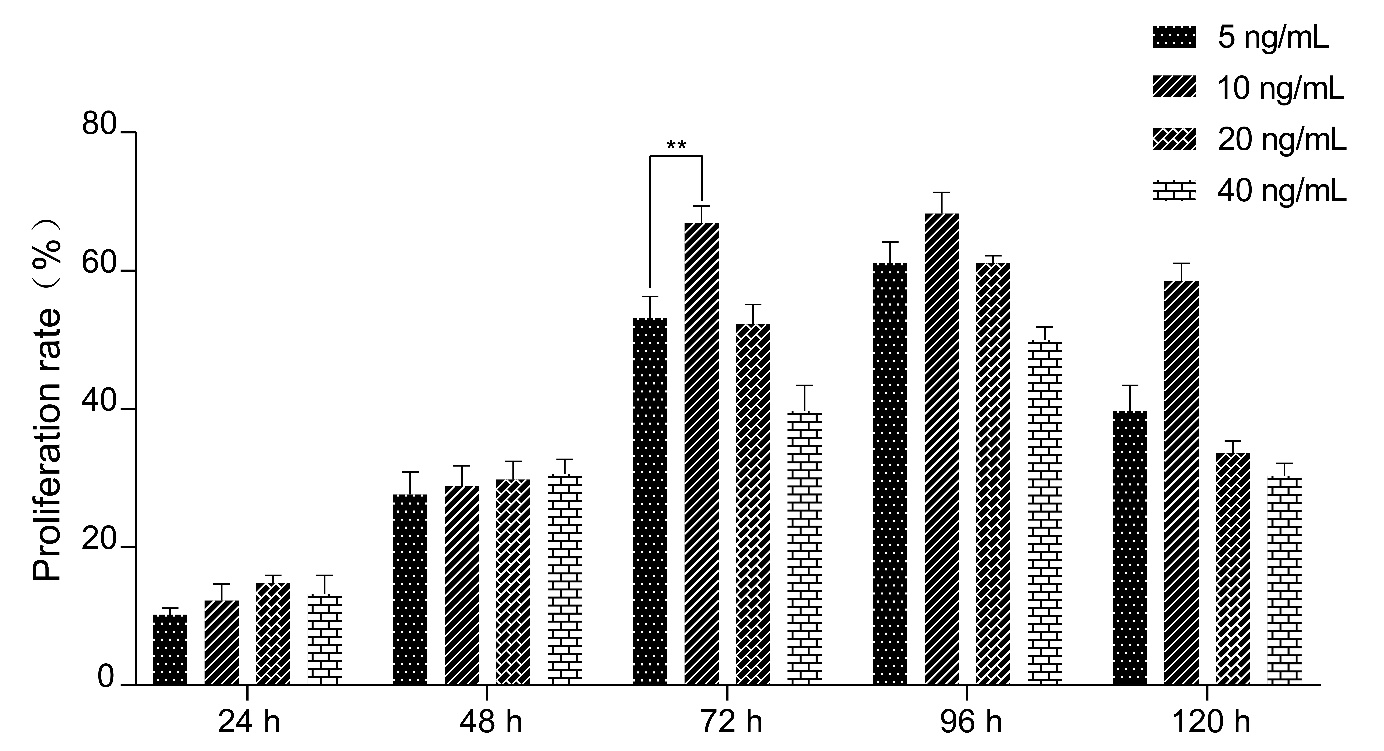


Figure S2. Dose- and time-dependent effects of TGF-β1 on LX-2 cell viability. Cells were treated with TGF-β1 at concentrations of 5, 10, 20, and 40 ng/mL for 24, 48, 72, 96, and 120 h, respectively. Data are presented as mean ± SD (n = 3); **, significant difference at P < 0.01.
