# Supplementary material for: Cerevisterol from Ophiocordyceps sinensis fruiting bodies against liver fibrosis
Source: Front Pharmacol. 2026 Jul 8;17:1825109. doi: 10.3389/fphar.2026.1825109 (PMC13388481; doi:10.3389/fphar.2026.1825109)
Supplement: Supplementary file 1 [file DataSheet2.zip › Raw data/Supplementary Materials/Figure S3.docx]

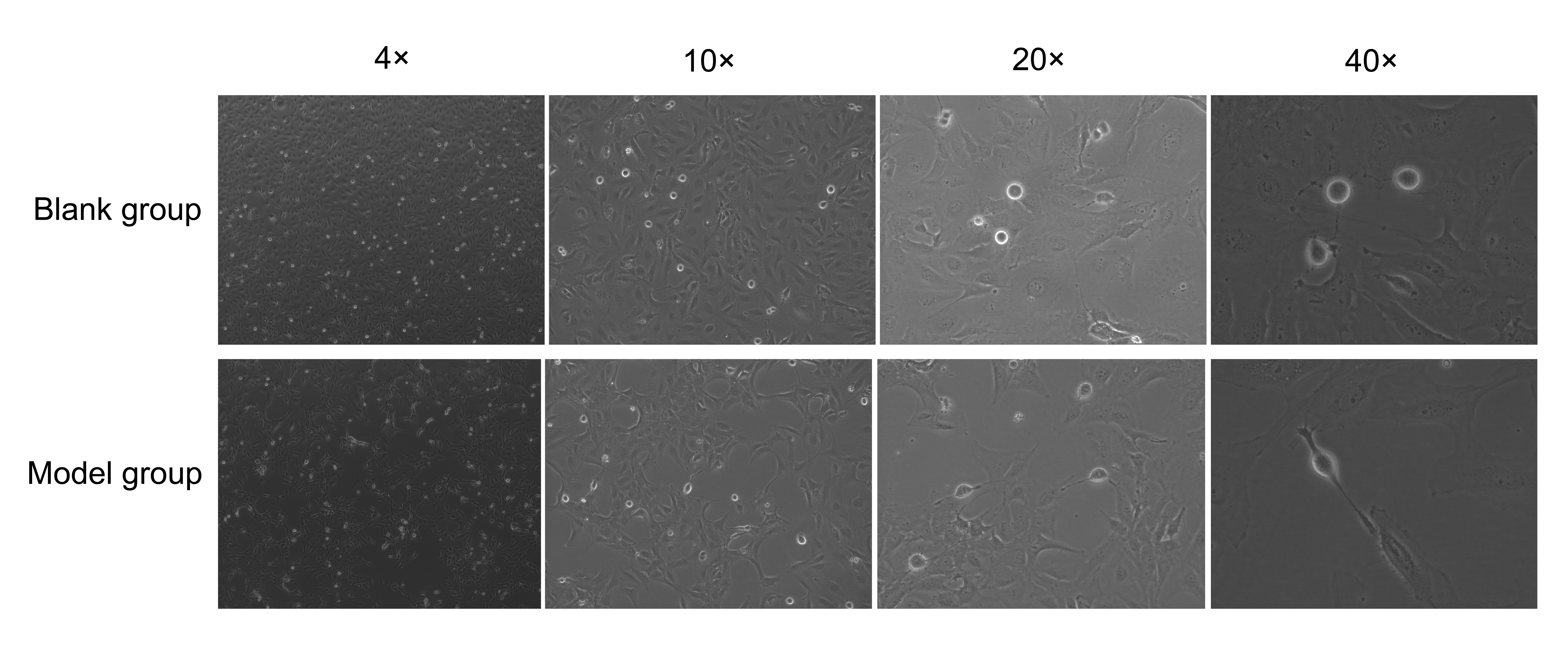


Figure S3. Morphological changes in LX-2 cells following TGF-β1-induced activation: comparison between untreated control cells (Blank group) and cells cultured with TGF-β1 (10 ng/mL) for 72 h (Model group).
