# Supplementary figures and images for: Cerevisterol from Ophiocordyceps sinensis fruiting bodies against liver fibrosis
Source: Front Pharmacol. 2026 Jul 8;17:1825109. doi: 10.3389/fphar.2026.1825109 (PMC13388481; doi:10.3389/fphar.2026.1825109)

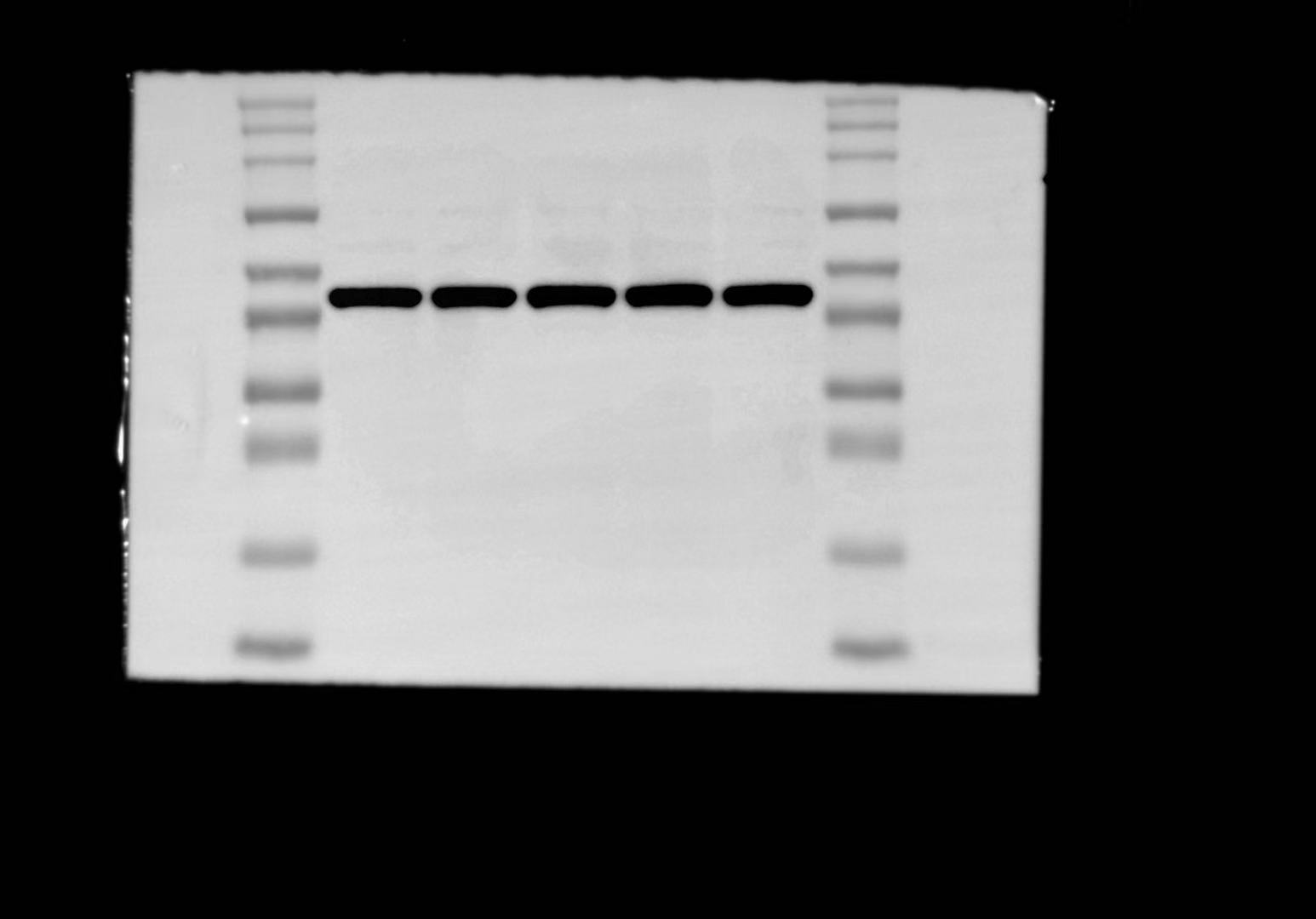

Supplement: Supplementary file 1 [file DataSheet2.zip › Raw data/Fig 5:Western blot membrane image/actin.jpg]

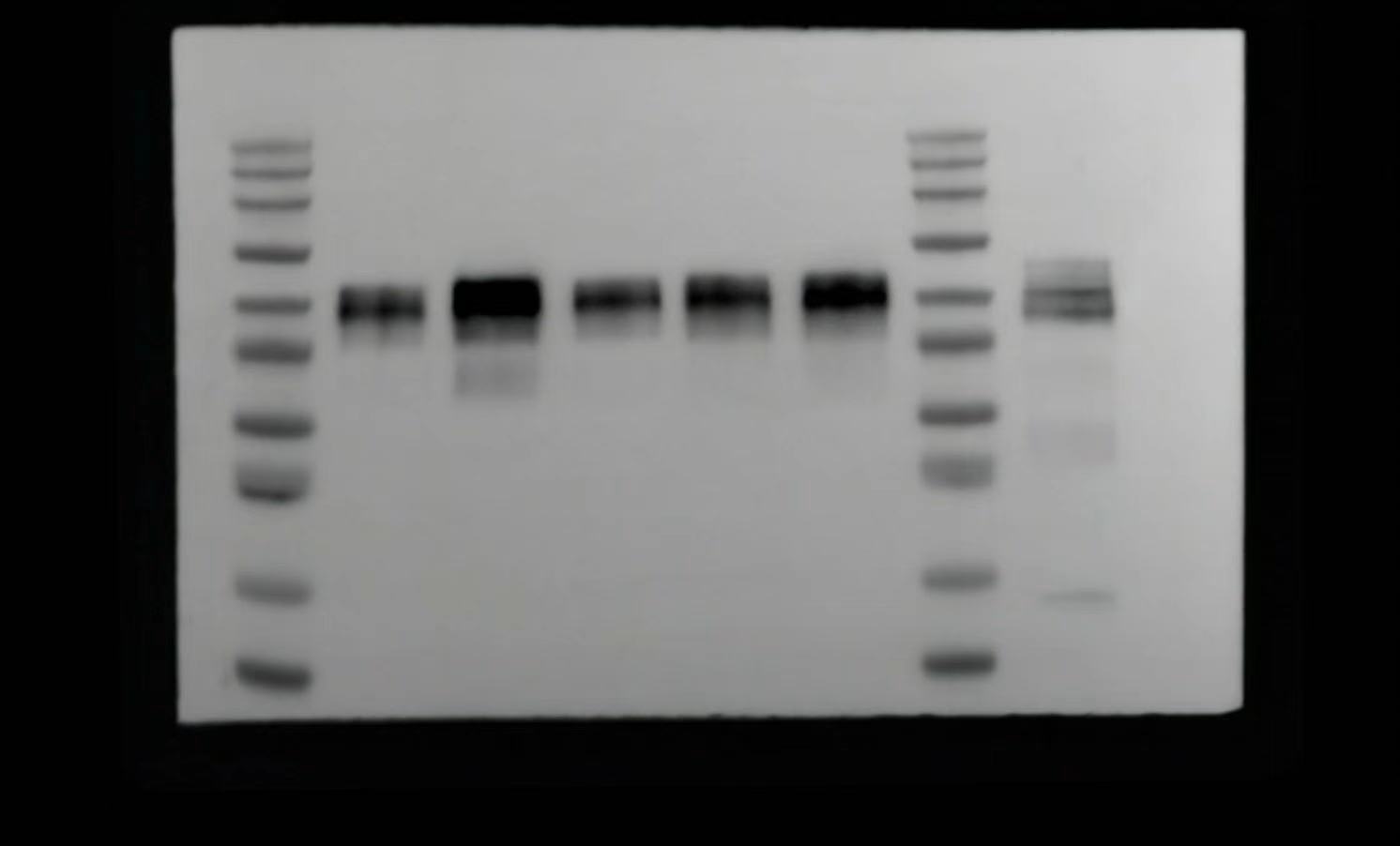

Supplement: Supplementary file 1 [file DataSheet2.zip › Raw data/Fig 5:Western blot membrane image/smad3.jpg]

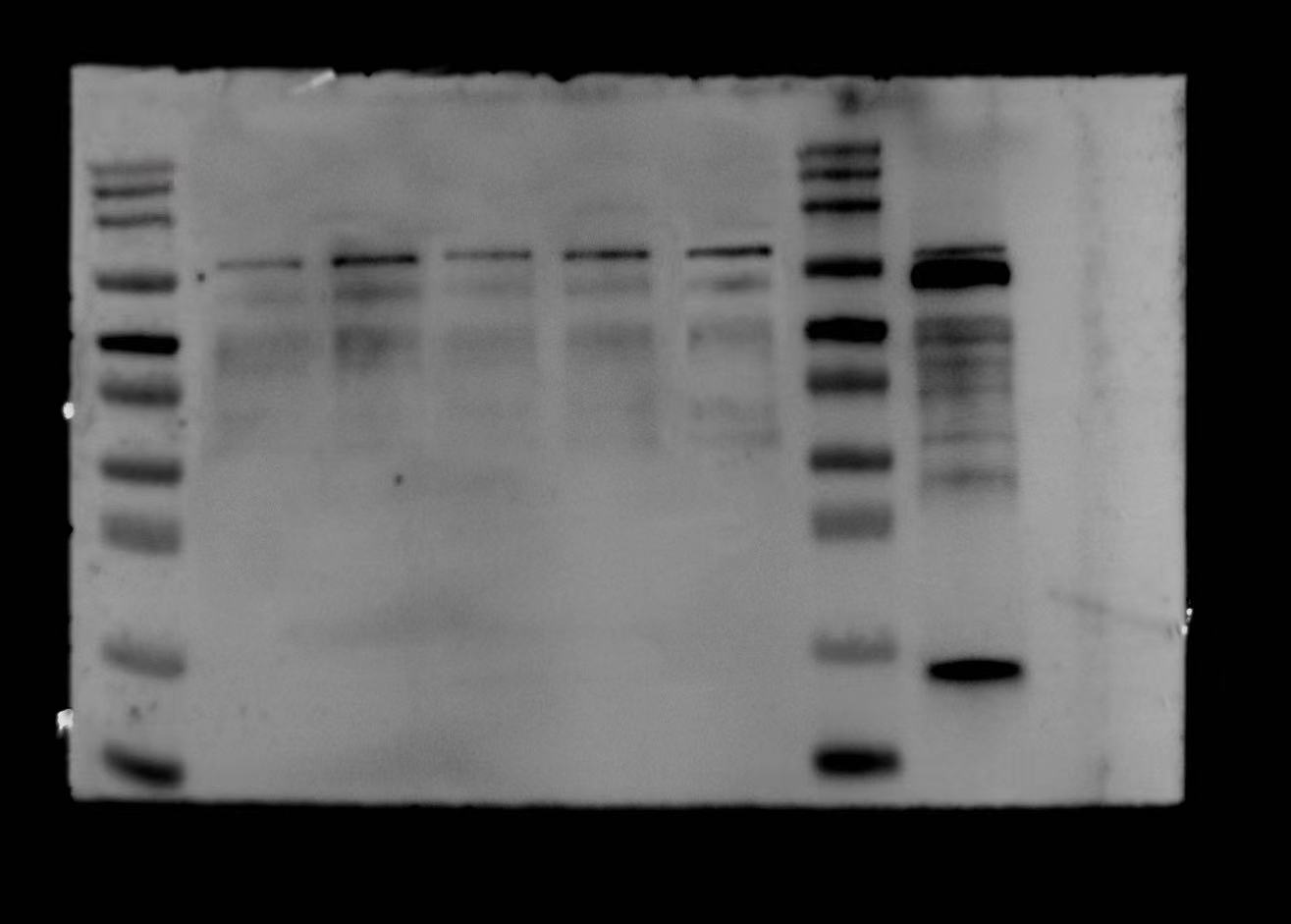

Supplement: Supplementary file 1 [file DataSheet2.zip › Raw data/Fig 5:Western blot membrane image/smad4.jpg]

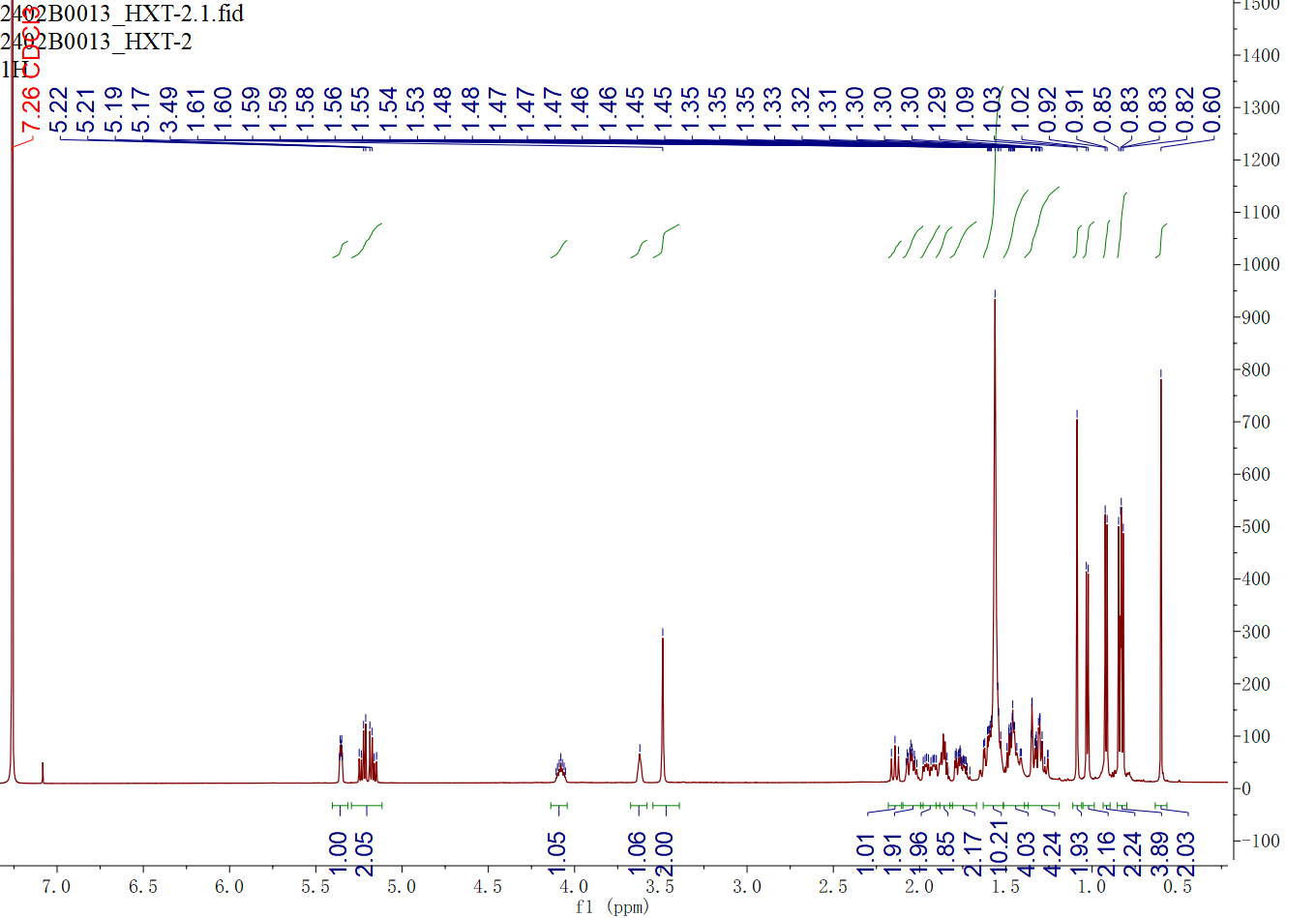

Supplement: Supplementary file 1 [file DataSheet2.zip › Raw data/Supplementary Materials/Figure S1:1H NMR and 13C NMR spectra/Fig S1A-Original image.tiff]

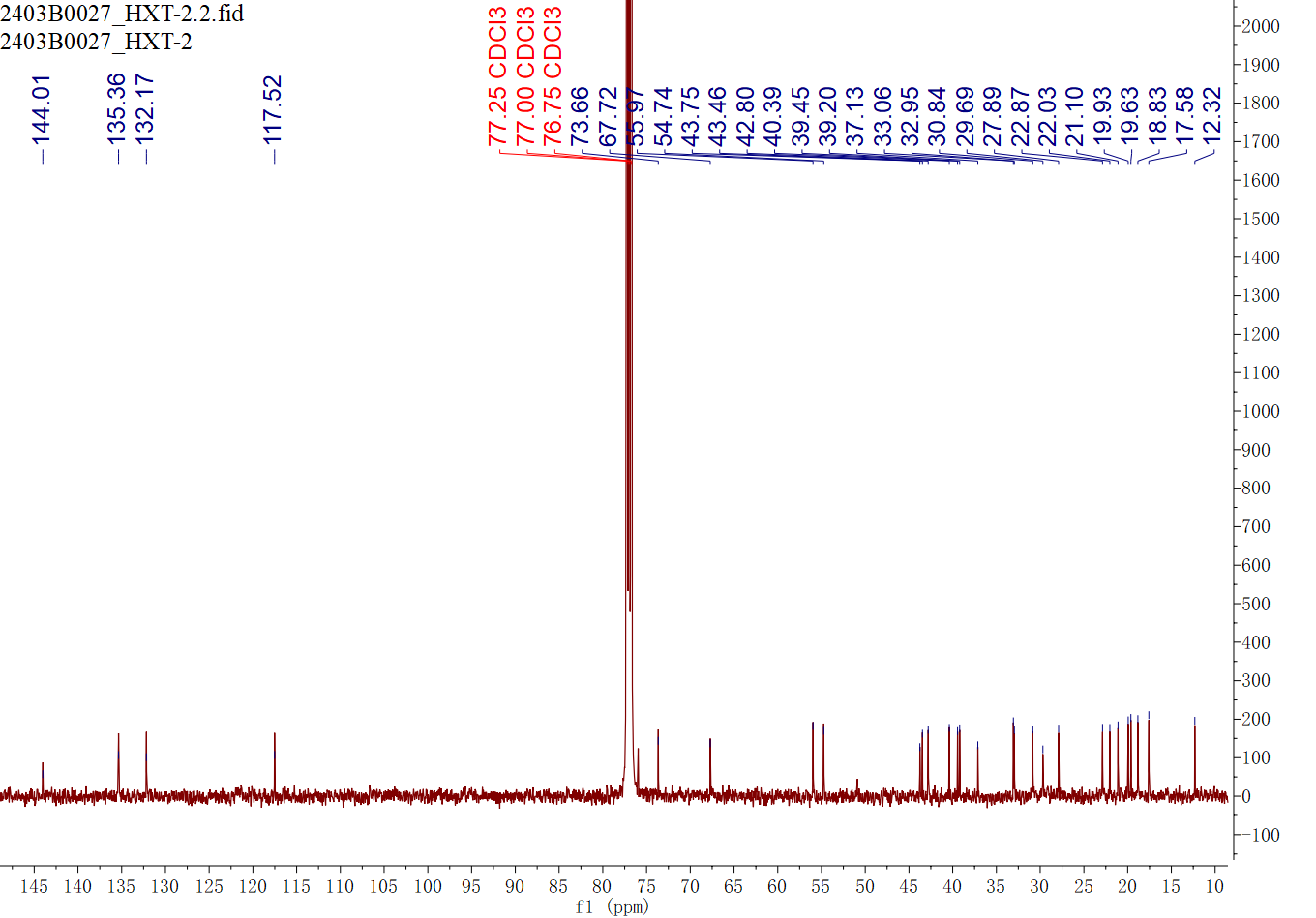

Supplement: Supplementary file 1 [file DataSheet2.zip › Raw data/Supplementary Materials/Figure S1:1H NMR and 13C NMR spectra/Fig S1B-Original image.tiff]

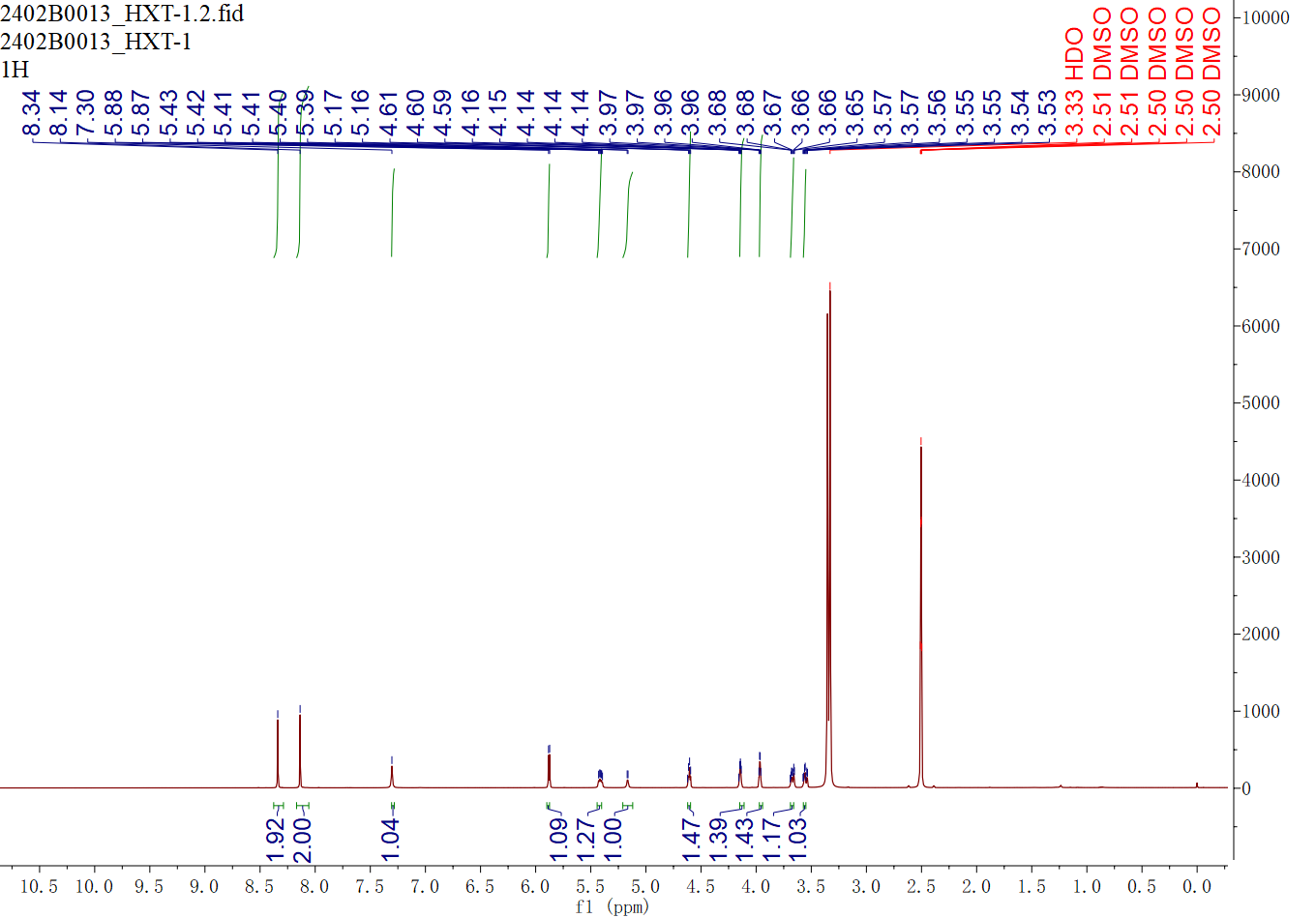

Supplement: Supplementary file 1 [file DataSheet2.zip › Raw data/Supplementary Materials/Figure S1:1H NMR and 13C NMR spectra/Fig S1C-Original image.tiff]

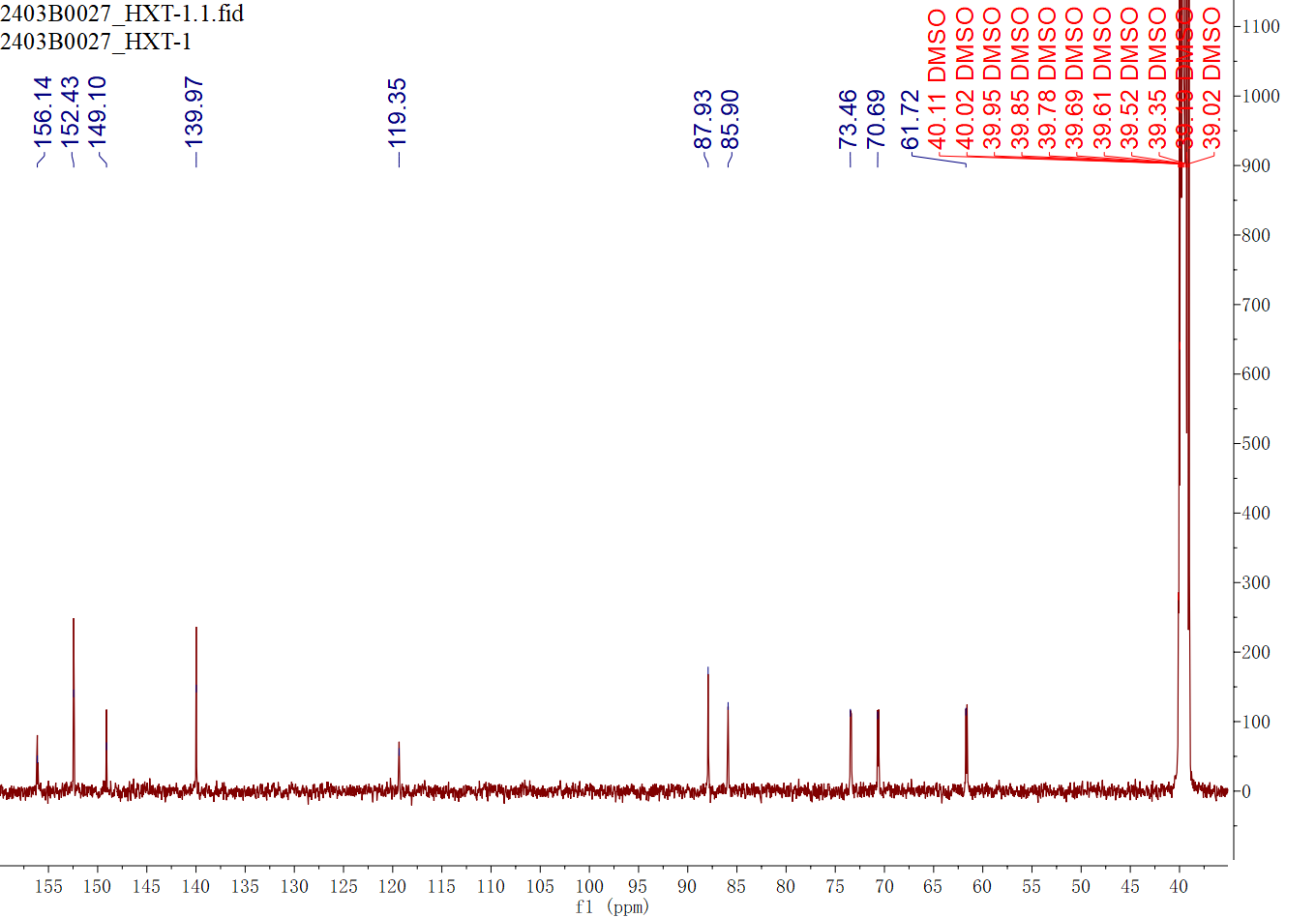

Supplement: Supplementary file 1 [file DataSheet2.zip › Raw data/Supplementary Materials/Figure S1:1H NMR and 13C NMR spectra/Fig S1D-Original image.tiff]

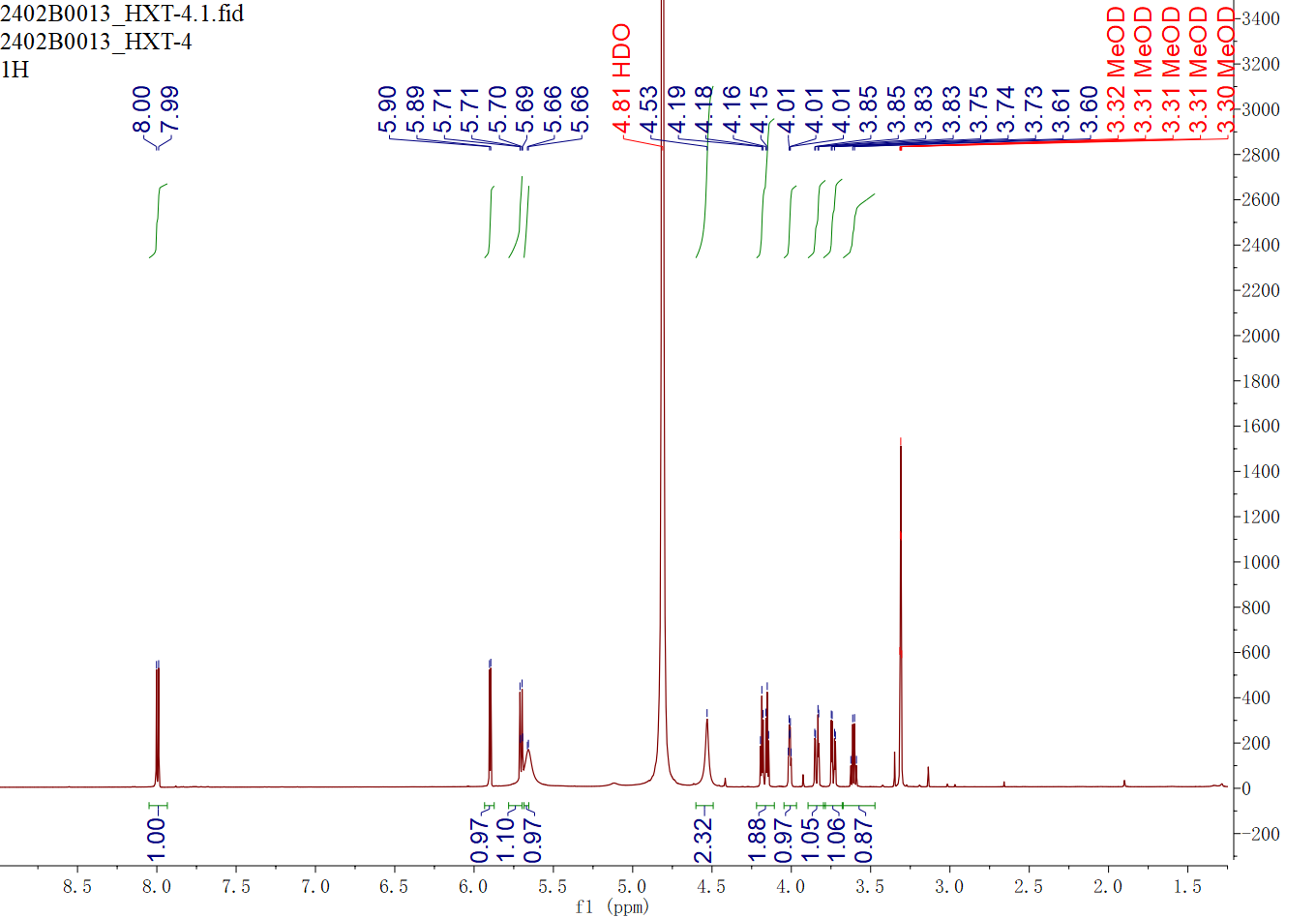

Supplement: Supplementary file 1 [file DataSheet2.zip › Raw data/Supplementary Materials/Figure S1:1H NMR and 13C NMR spectra/Fig S1E-Original image.tiff]

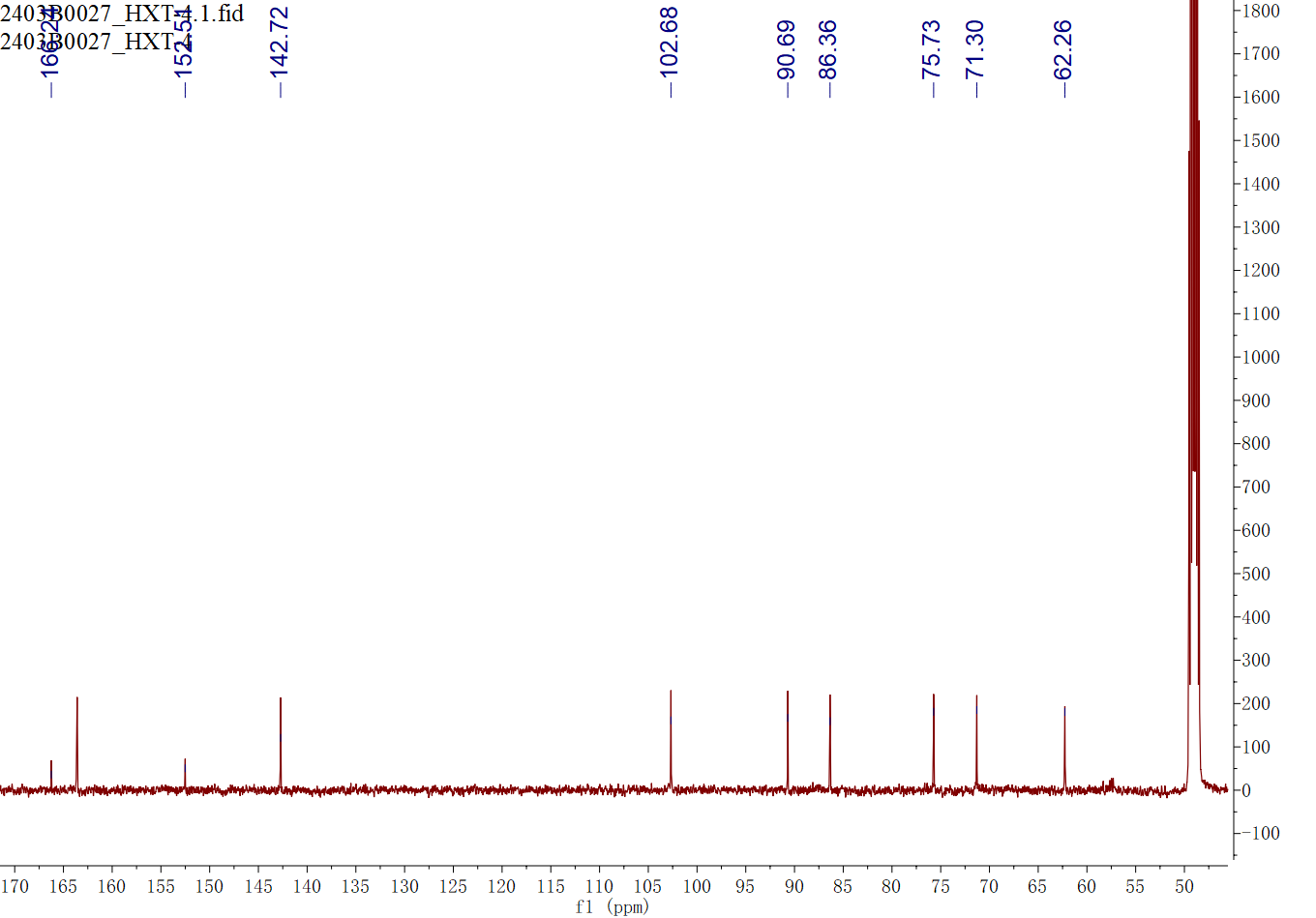

Supplement: Supplementary file 1 [file DataSheet2.zip › Raw data/Supplementary Materials/Figure S1:1H NMR and 13C NMR spectra/Fig S1F-Original image.tiff]

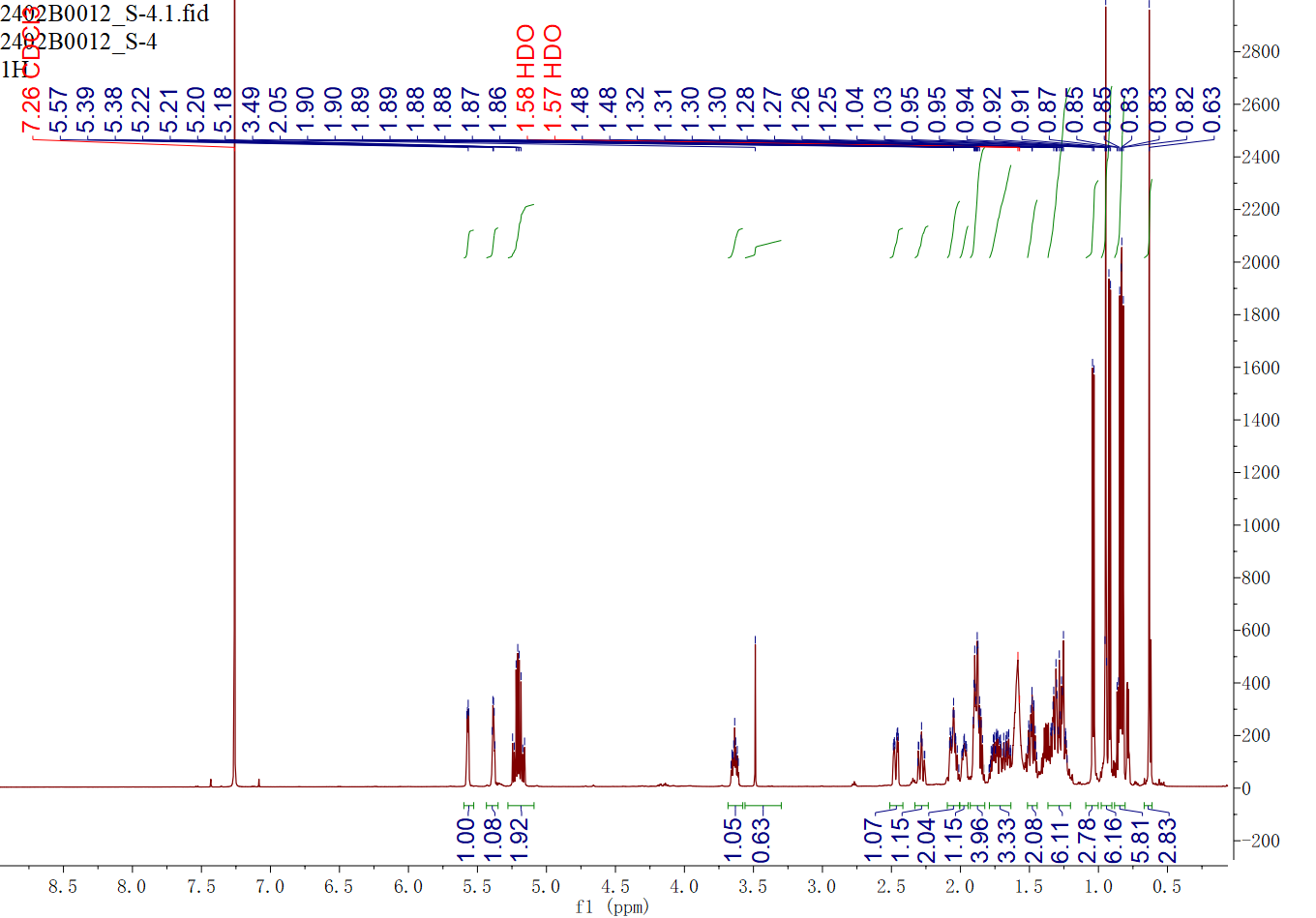

Supplement: Supplementary file 1 [file DataSheet2.zip › Raw data/Supplementary Materials/Figure S1:1H NMR and 13C NMR spectra/Fig S1G-Original image.tiff]

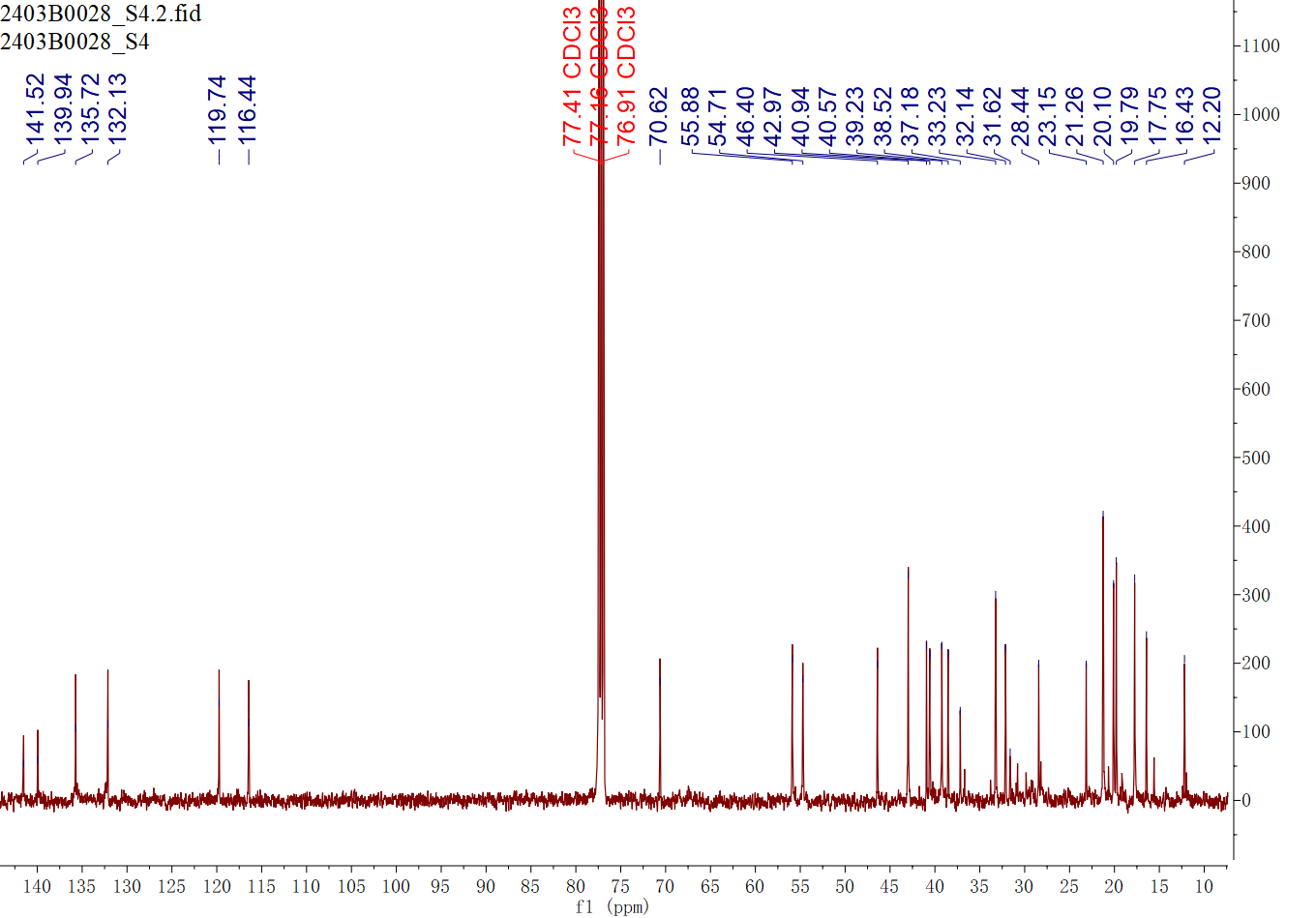

Supplement: Supplementary file 1 [file DataSheet2.zip › Raw data/Supplementary Materials/Figure S1:1H NMR and 13C NMR spectra/Fig S1H-Original image.tiff]

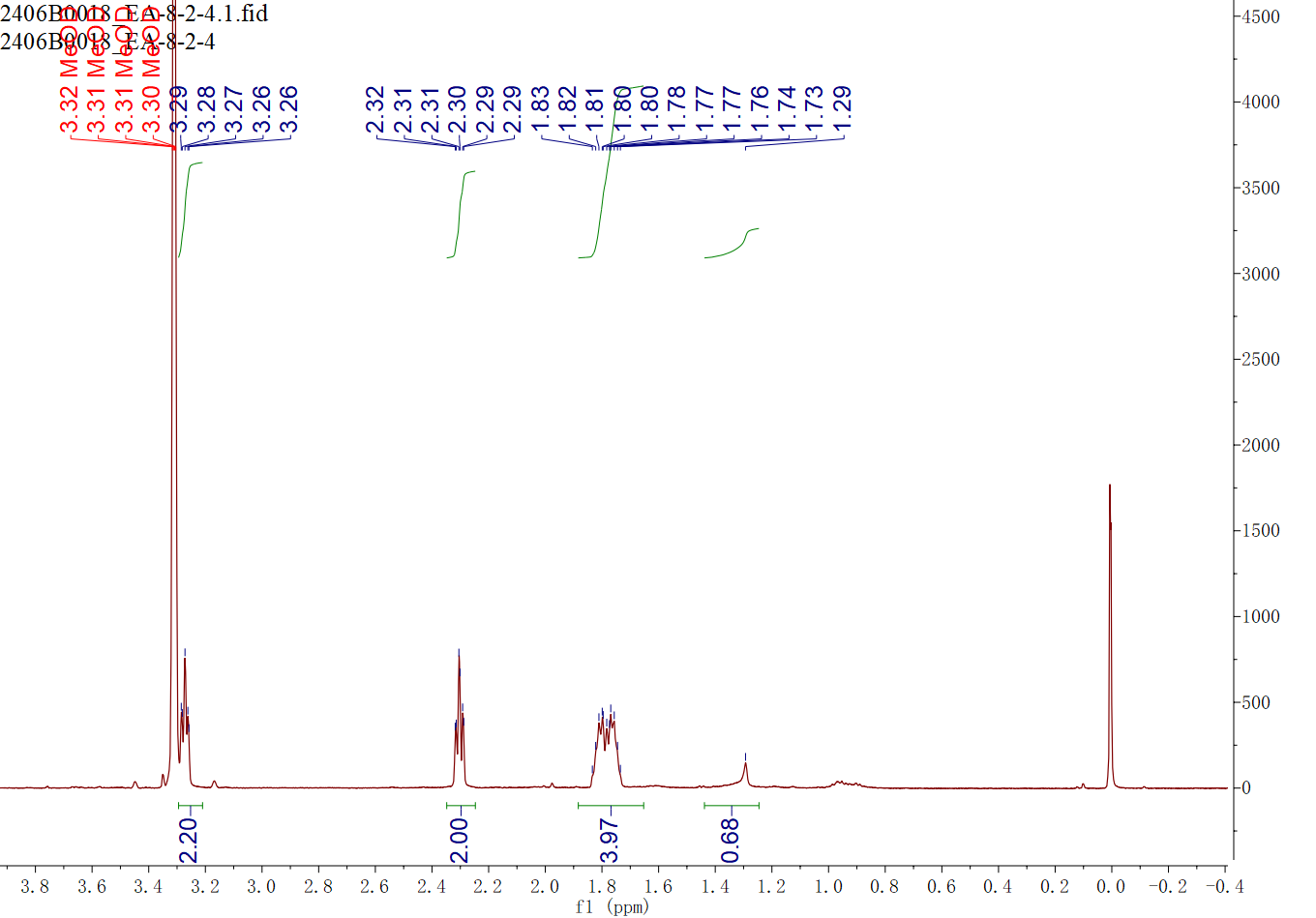

Supplement: Supplementary file 1 [file DataSheet2.zip › Raw data/Supplementary Materials/Figure S1:1H NMR and 13C NMR spectra/Fig S1I-Original image.tiff]

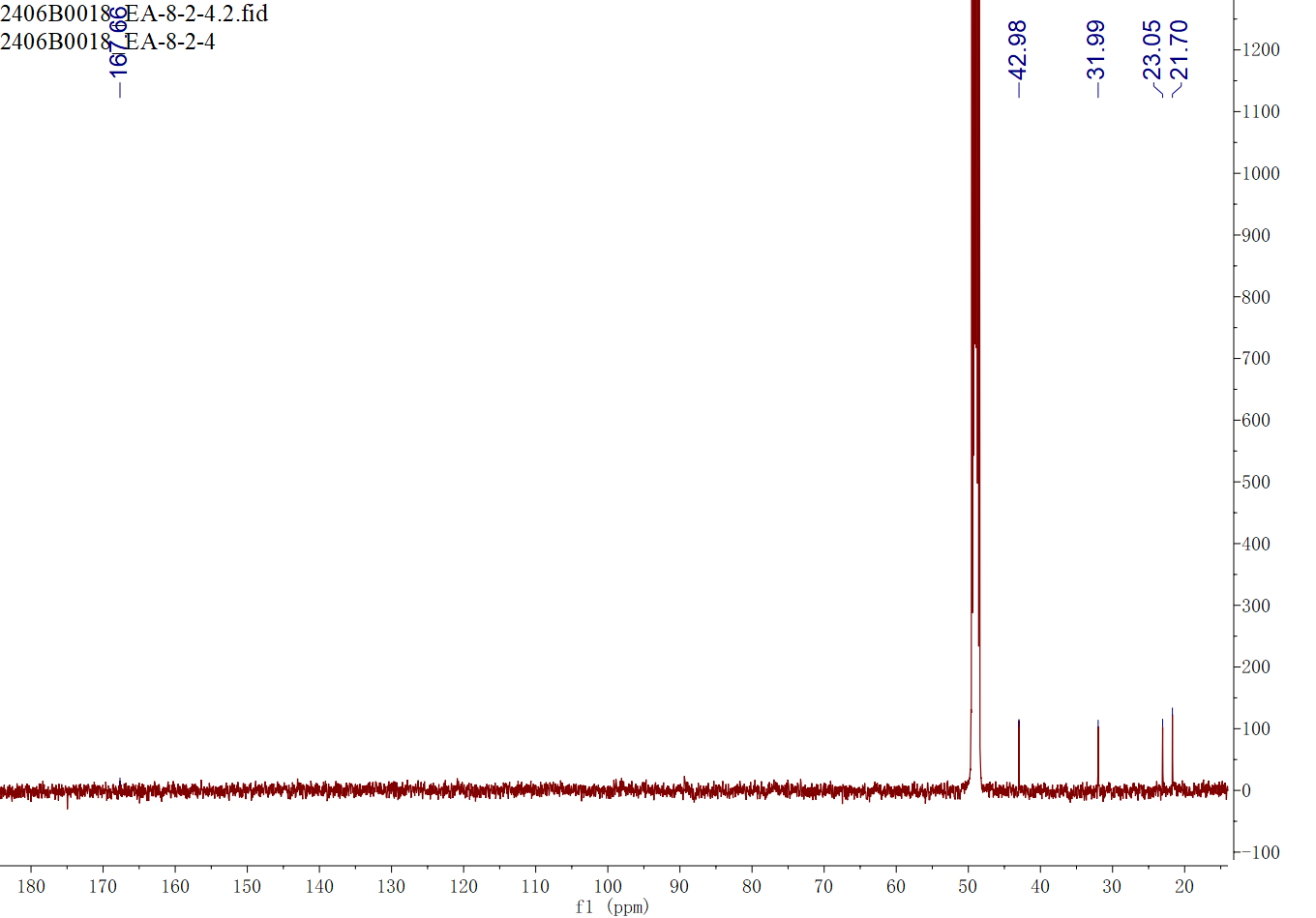

Supplement: Supplementary file 1 [file DataSheet2.zip › Raw data/Supplementary Materials/Figure S1:1H NMR and 13C NMR spectra/Fig S1J-Original image.tiff]

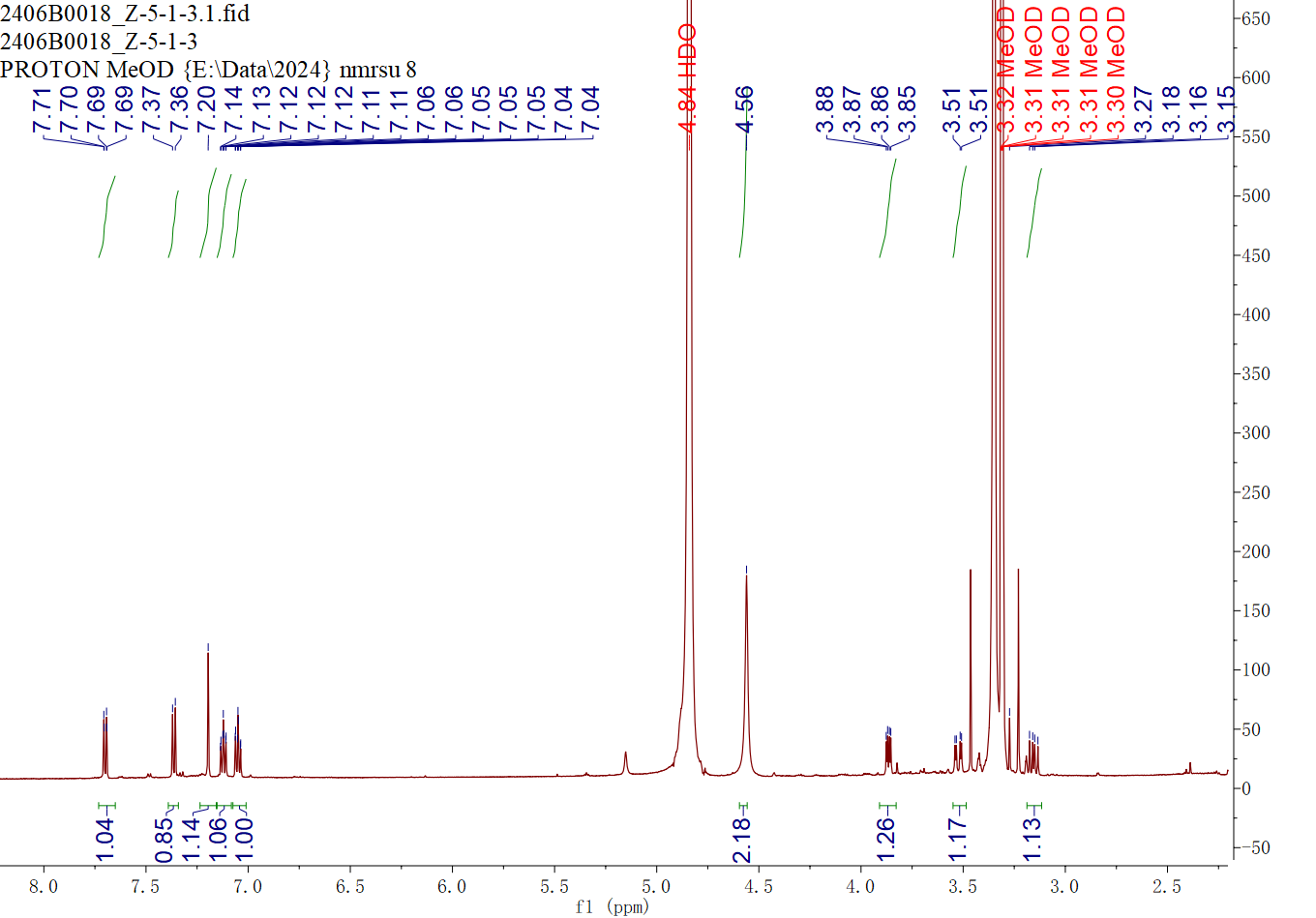

Supplement: Supplementary file 1 [file DataSheet2.zip › Raw data/Supplementary Materials/Figure S1:1H NMR and 13C NMR spectra/Fig S1K-Original image.tiff]

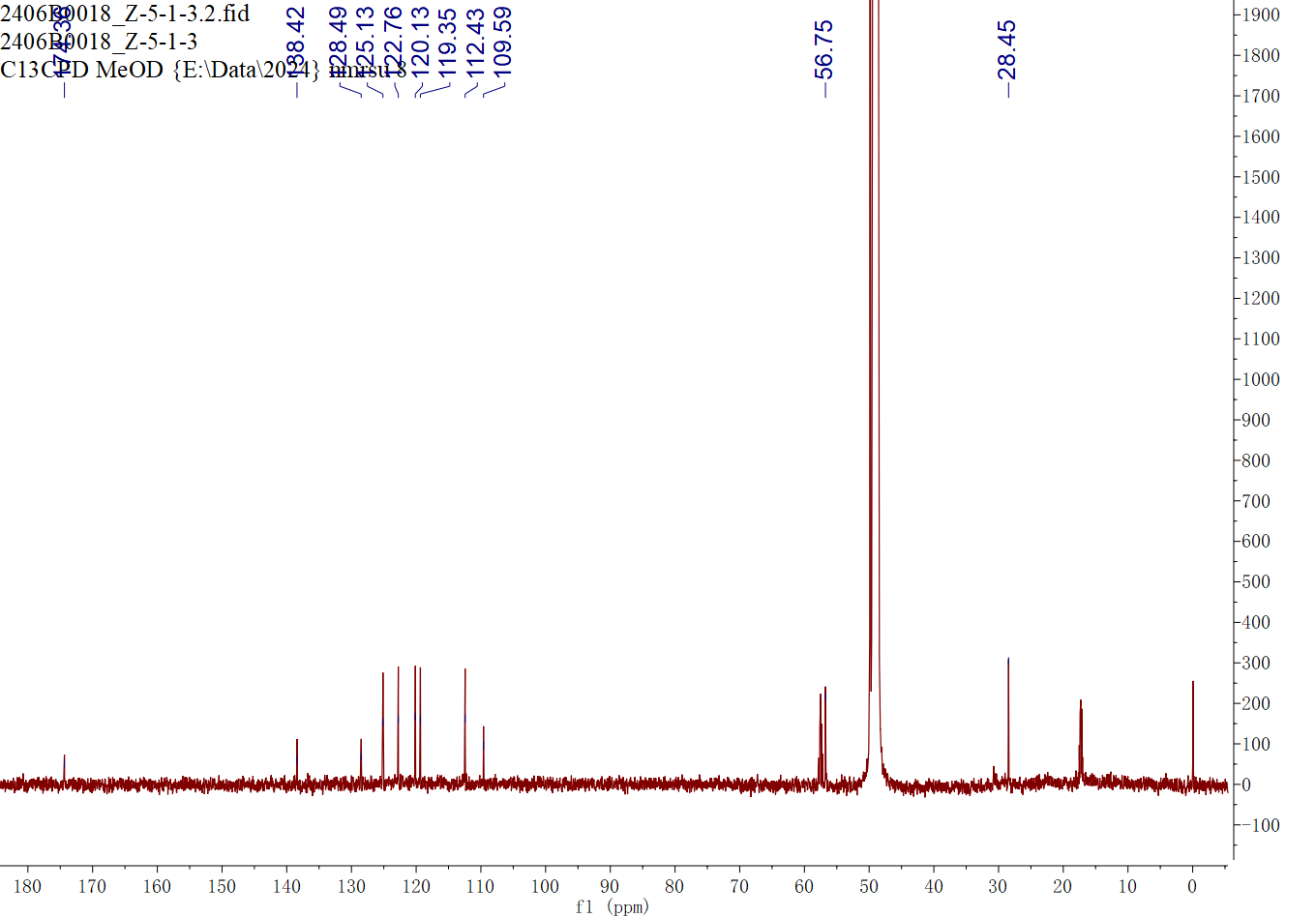

Supplement: Supplementary file 1 [file DataSheet2.zip › Raw data/Supplementary Materials/Figure S1:1H NMR and 13C NMR spectra/Fig S1L-Original image.tiff]

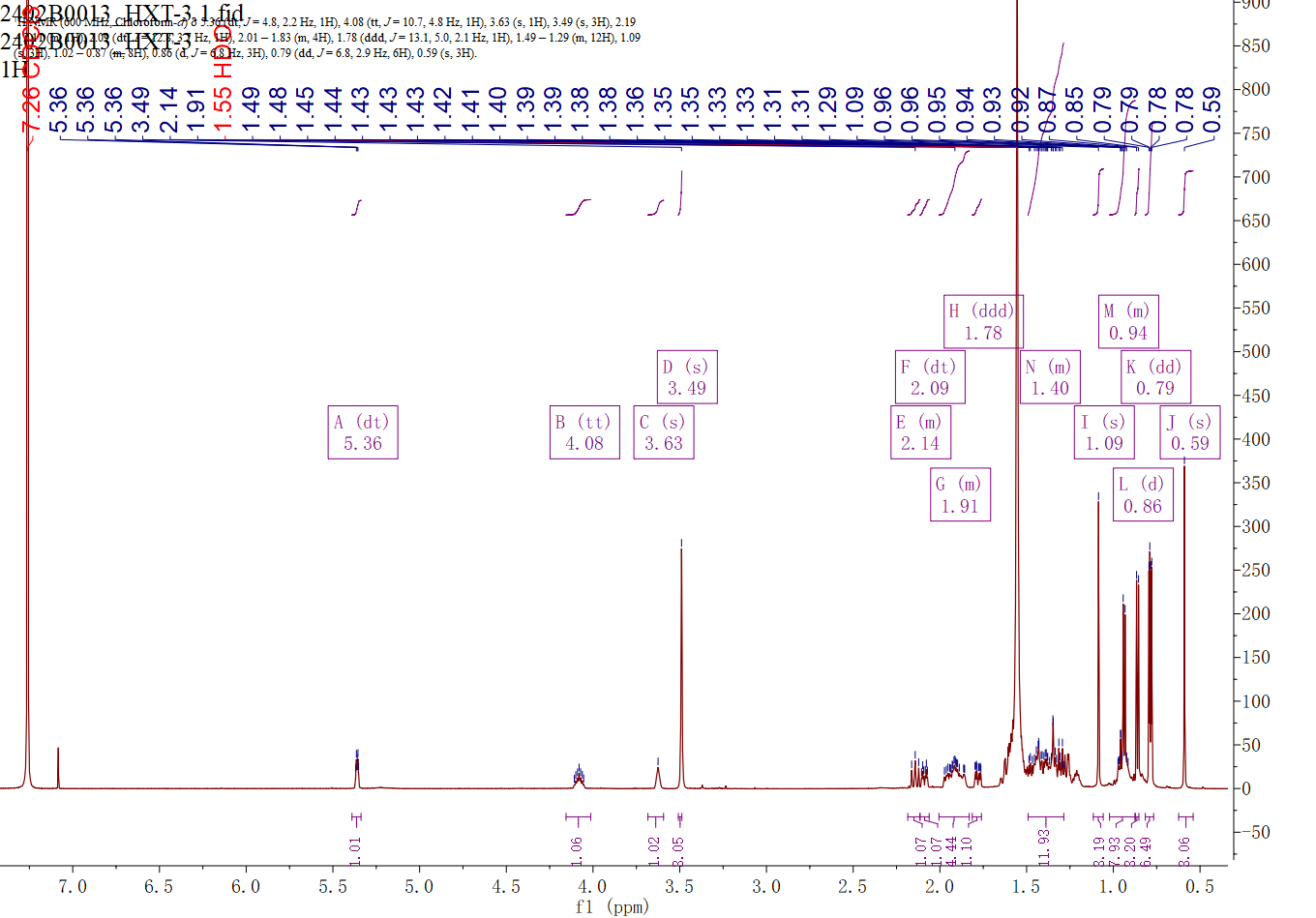

Supplement: Supplementary file 1 [file DataSheet2.zip › Raw data/Supplementary Materials/Figure S1:1H NMR and 13C NMR spectra/Fig S1M-Original image.tiff]

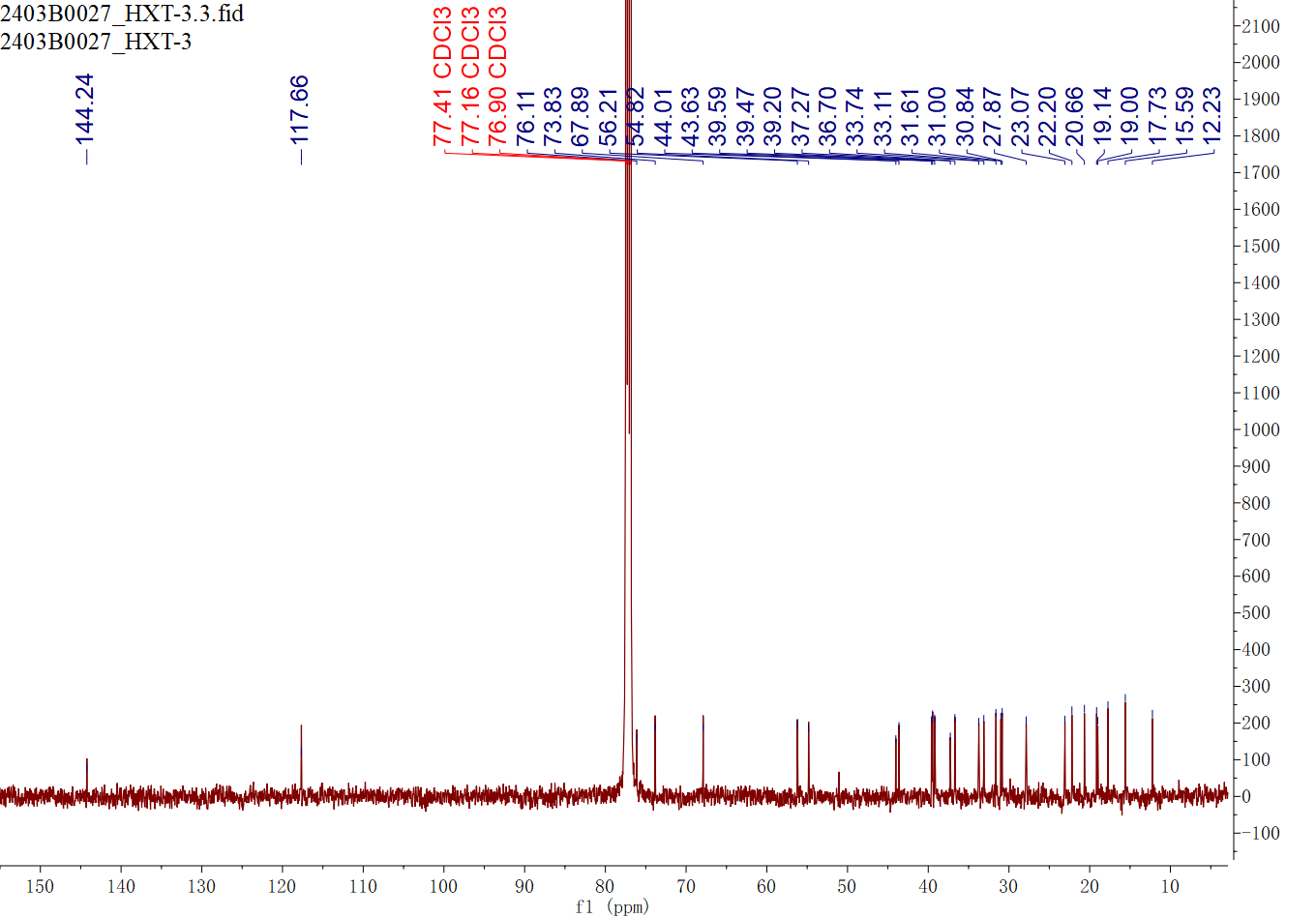

Supplement: Supplementary file 1 [file DataSheet2.zip › Raw data/Supplementary Materials/Figure S1:1H NMR and 13C NMR spectra/Fig S1N-Original image.tiff]
