# Supplementary material for: Cerevisterol from Ophiocordyceps sinensis fruiting bodies against liver fibrosis
Source: Front Pharmacol. 2026 Jul 8;17:1825109. doi: 10.3389/fphar.2026.1825109 (PMC13388481; doi:10.3389/fphar.2026.1825109)
Supplement: Supplementary file 2 [file DataSheet1.docx]

Supplementary Material

# Supplementary Figures and Tables

For more information on Supplementary Material and for details on the different file types accepted, please see [here](https://www.frontiersin.org/guidelines/author-guidelines#supplementary-material).

## Supplementary Figures

**A B**

**
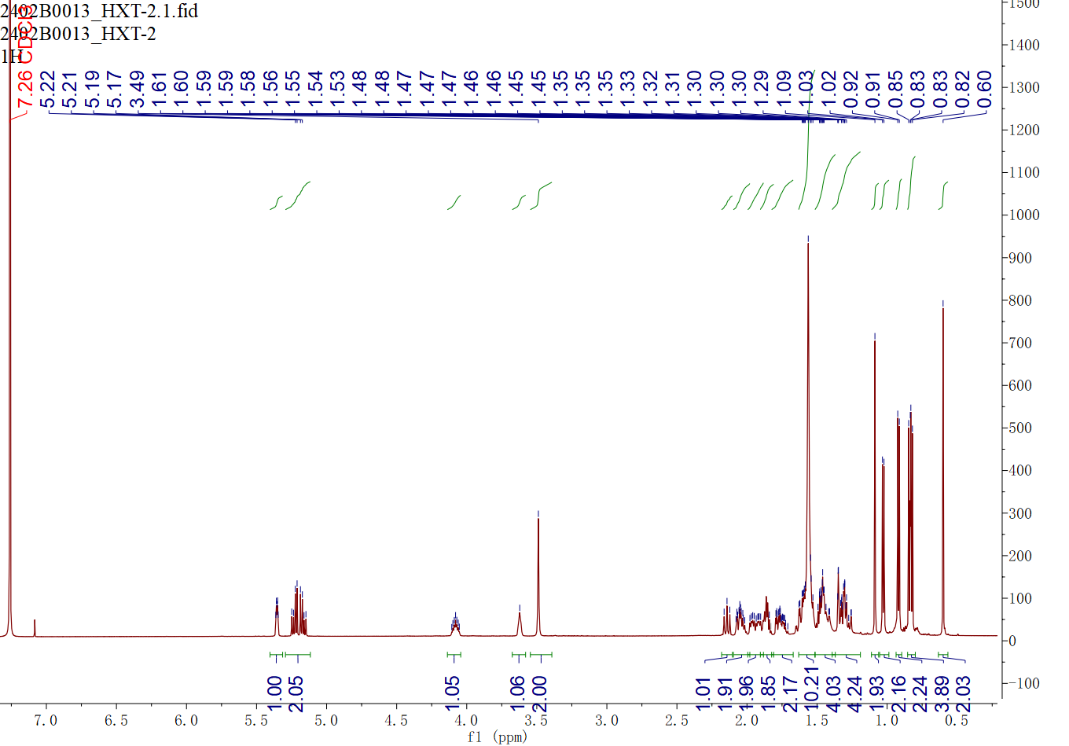

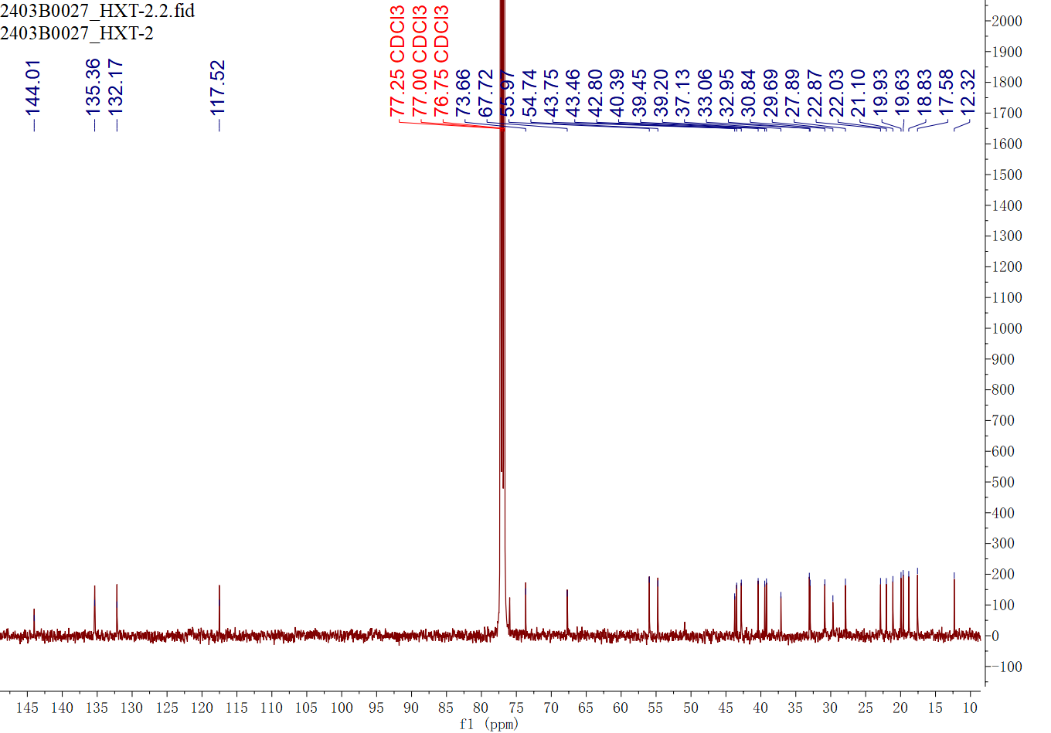
**

**C D**

**
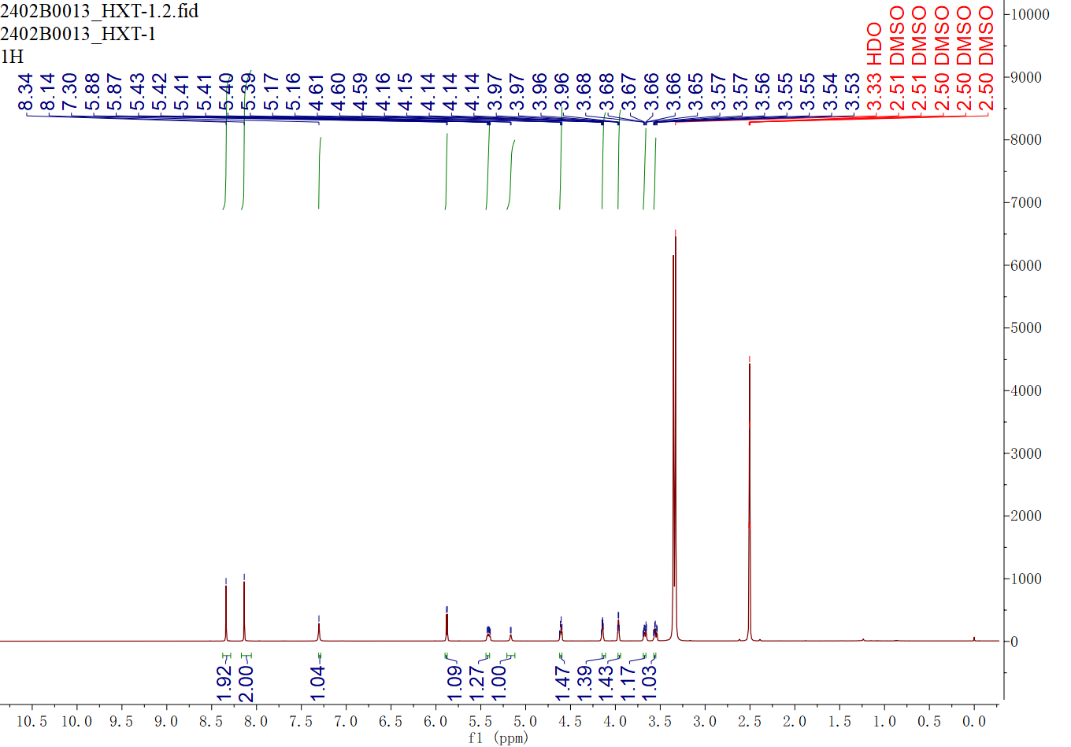

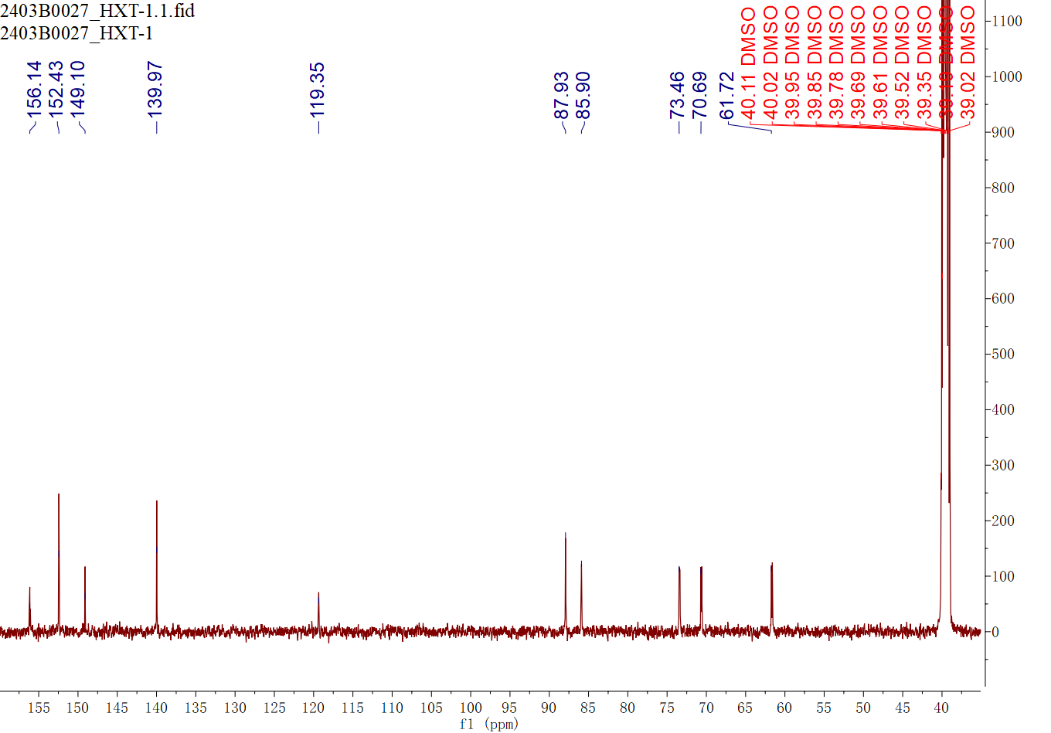
**

**E F**

**
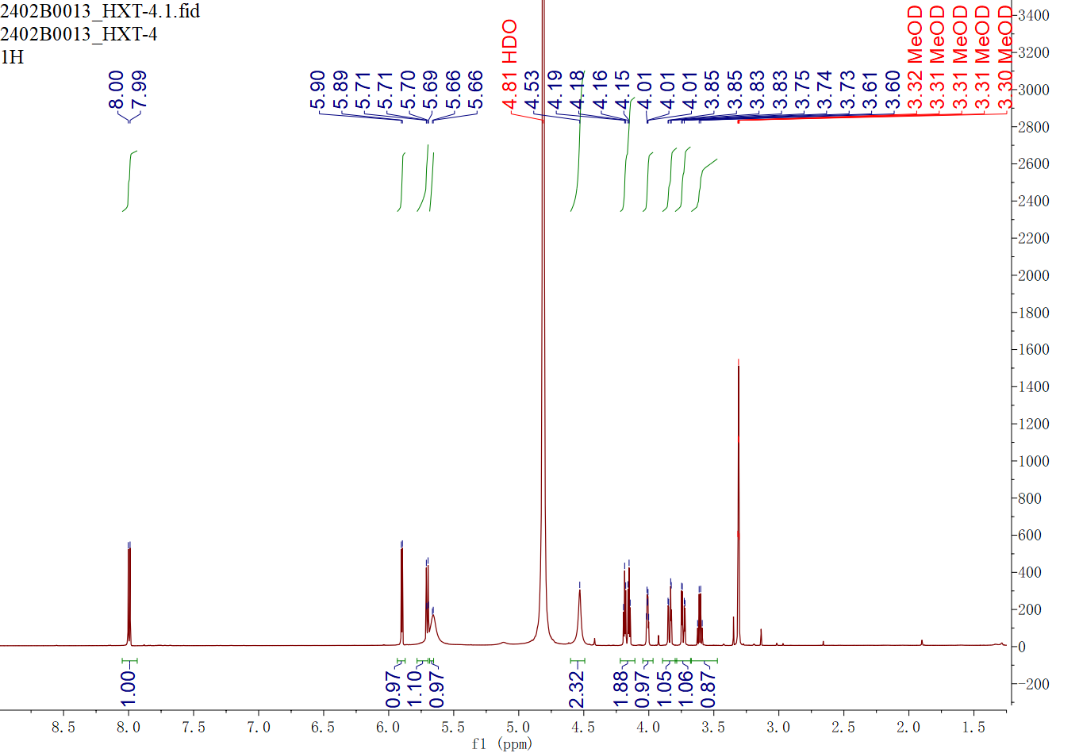

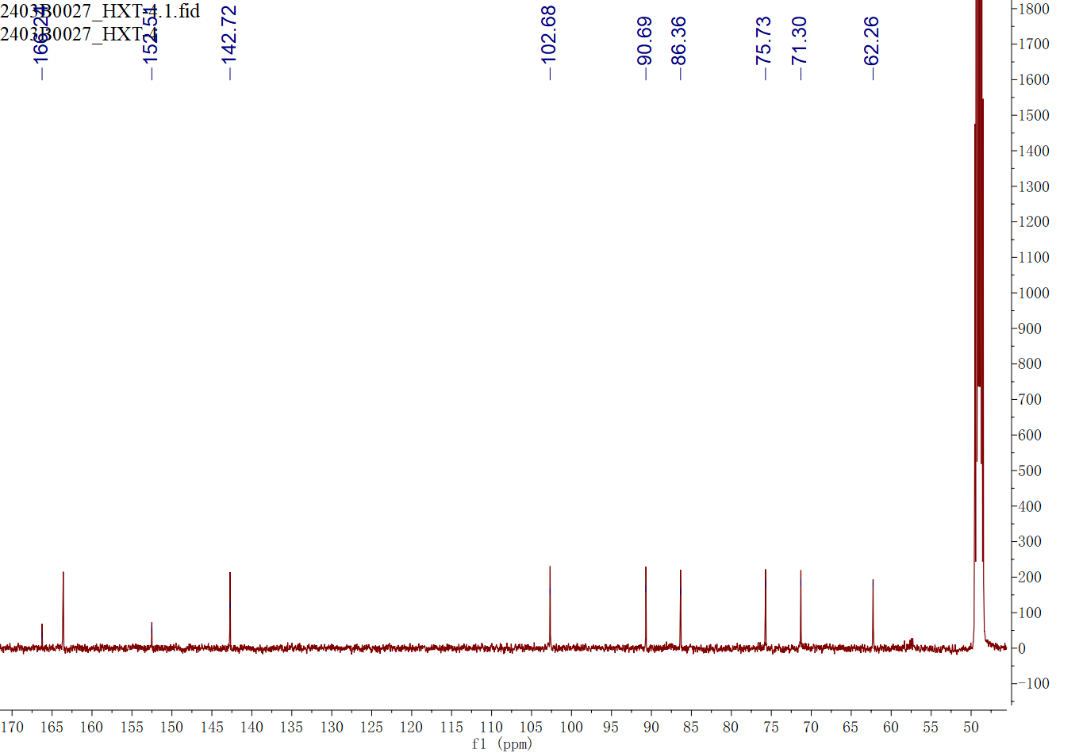
**

**G H**

**
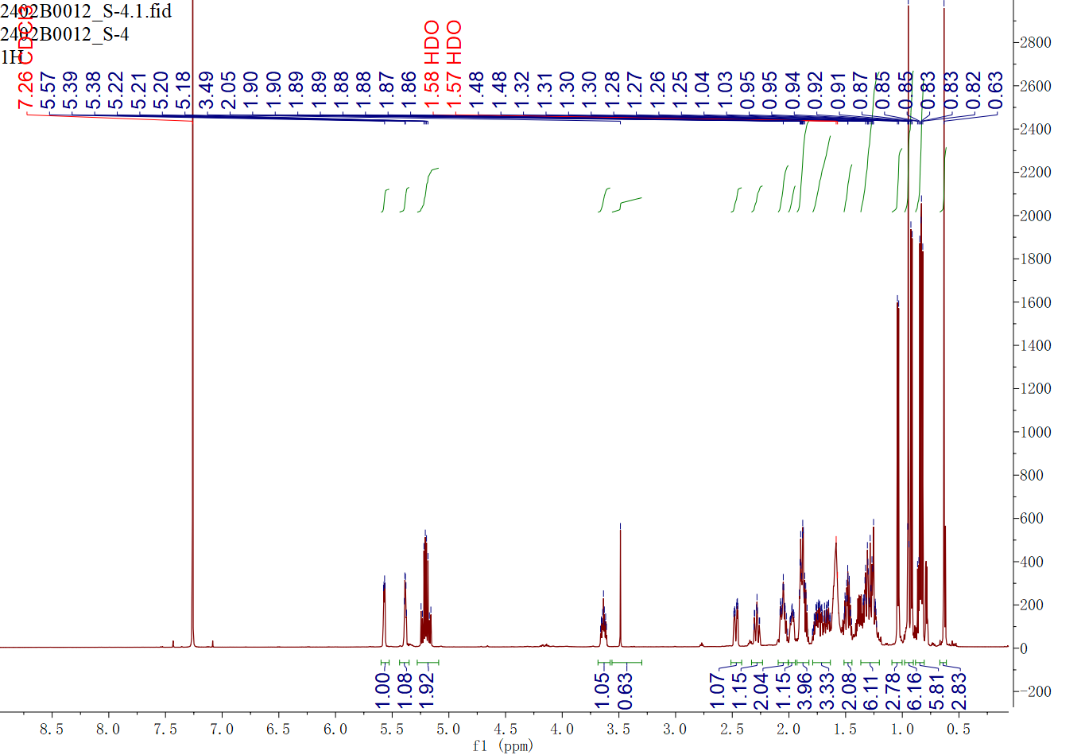

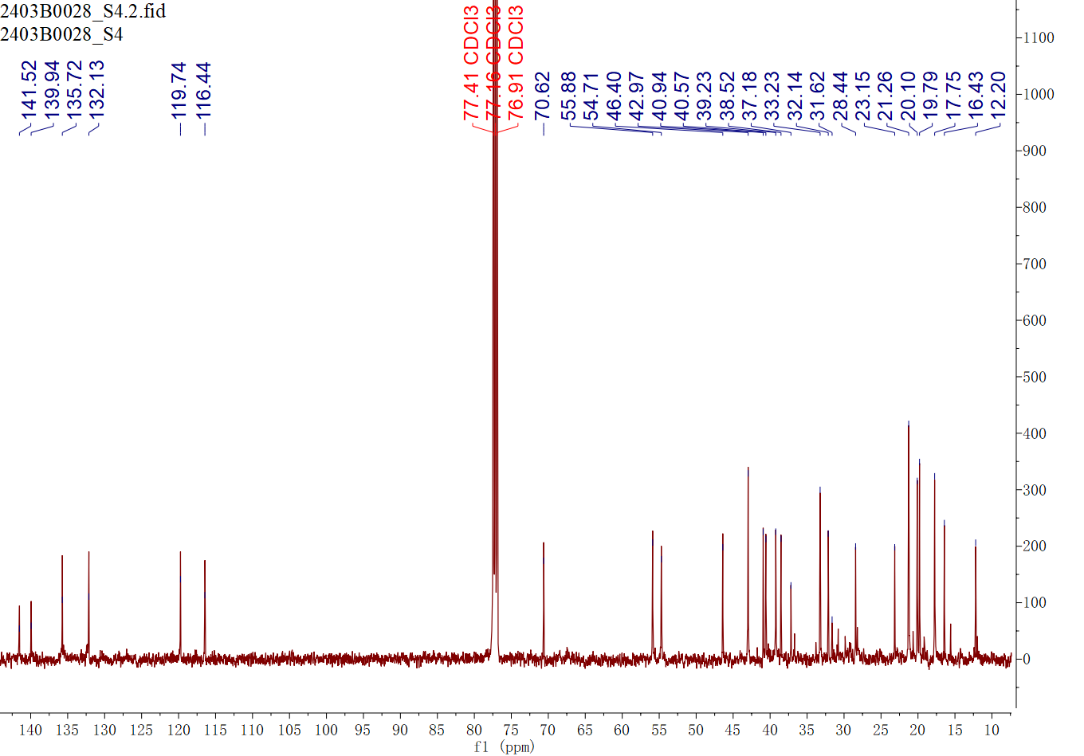
**

**I J**

**
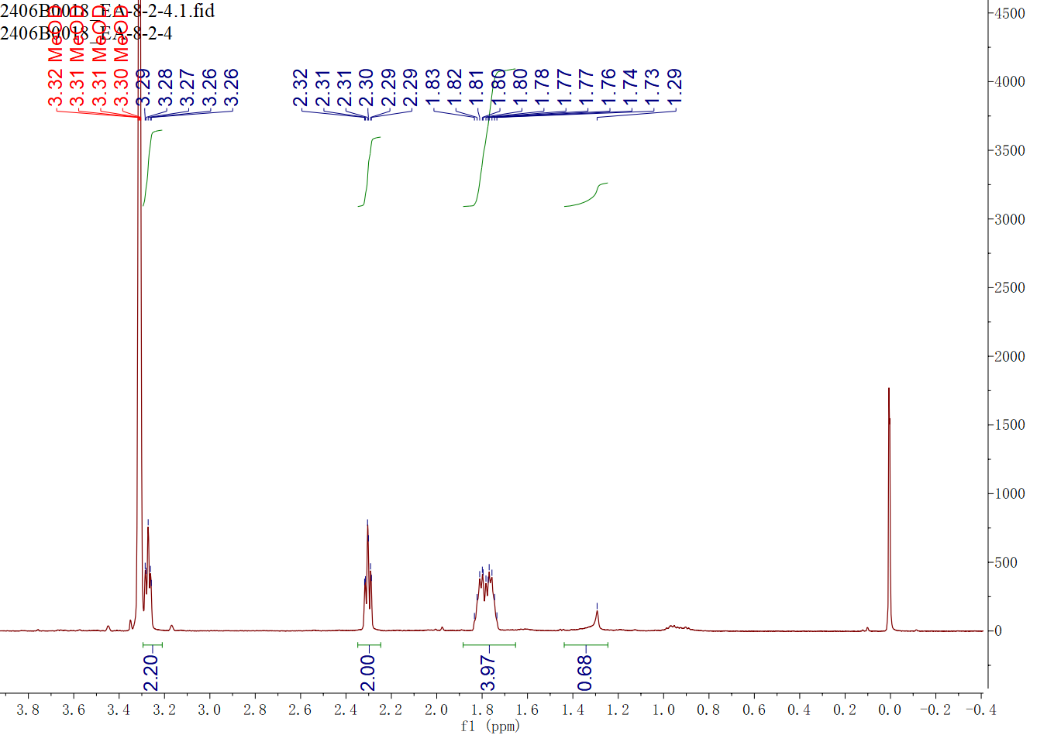

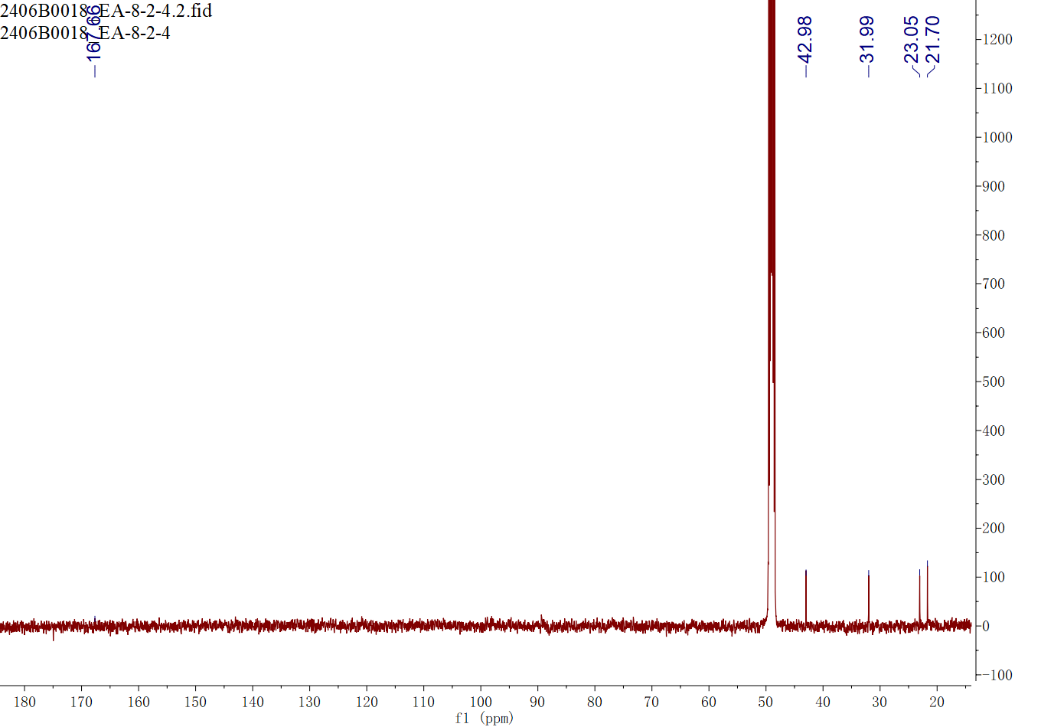
**

**K L**

**
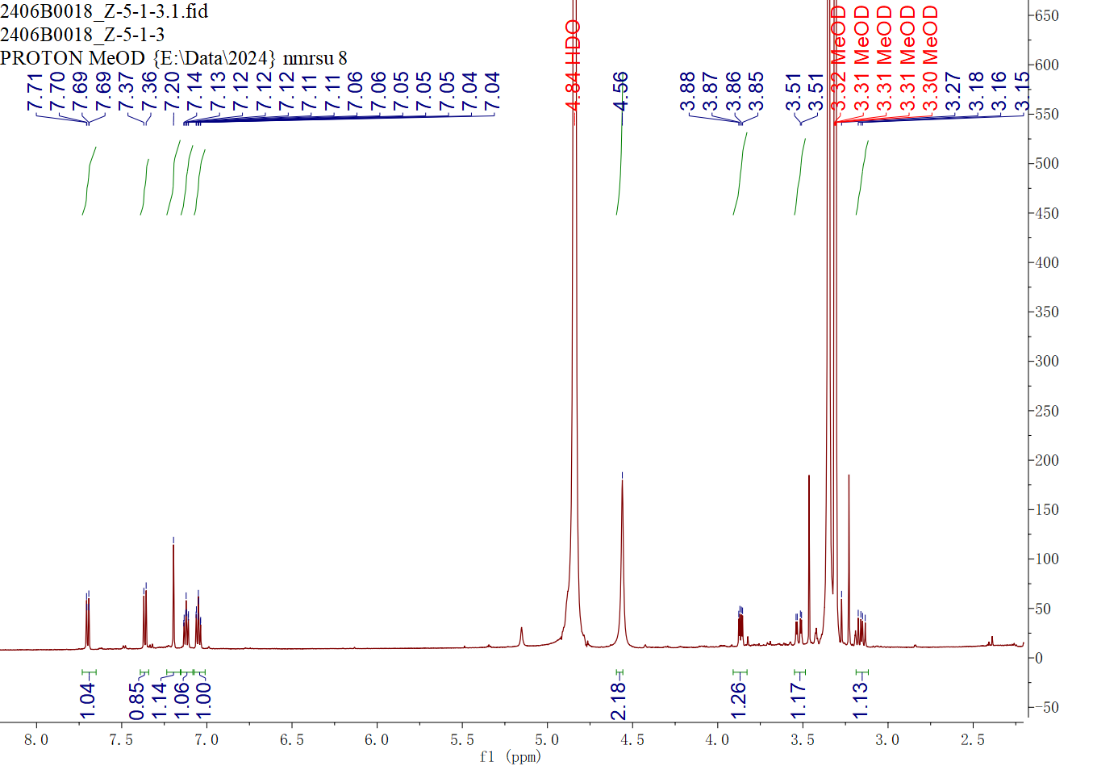

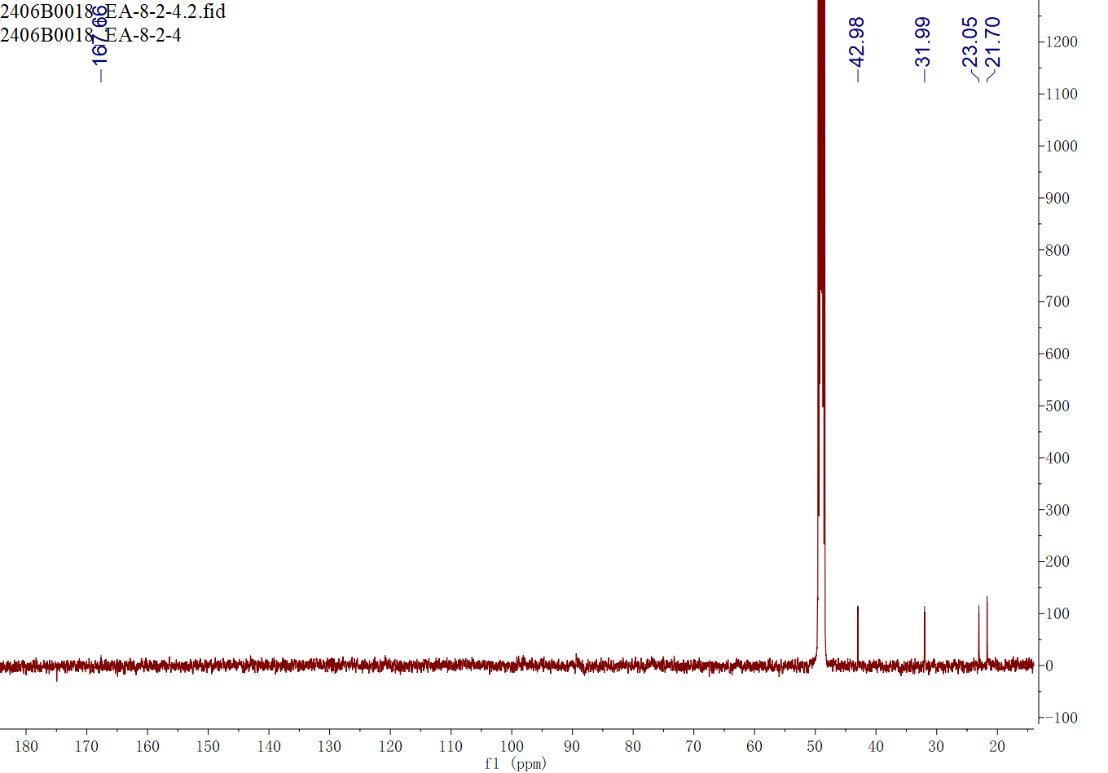
**

**M N**

**
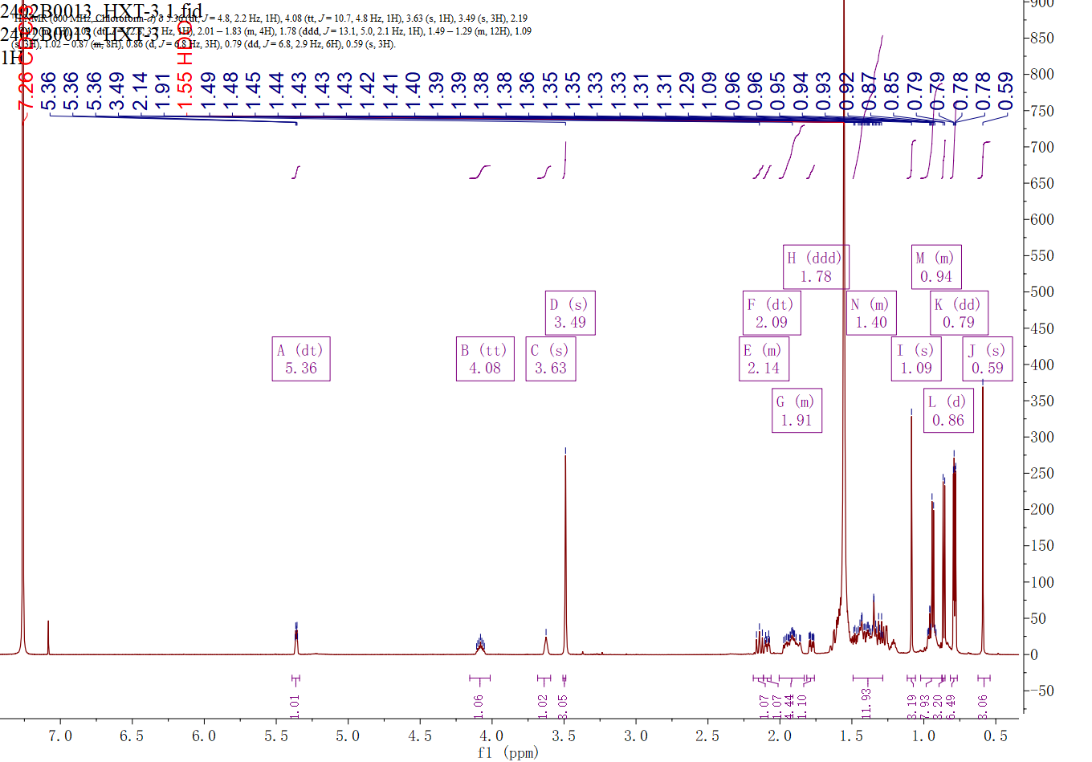

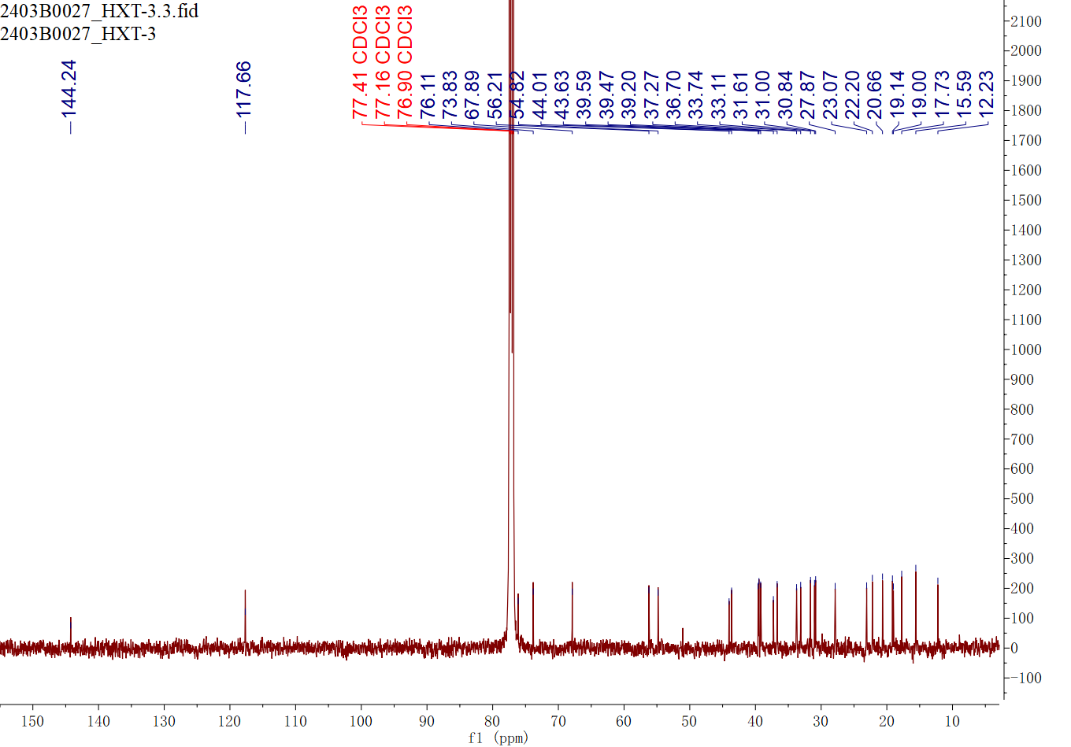
**

**
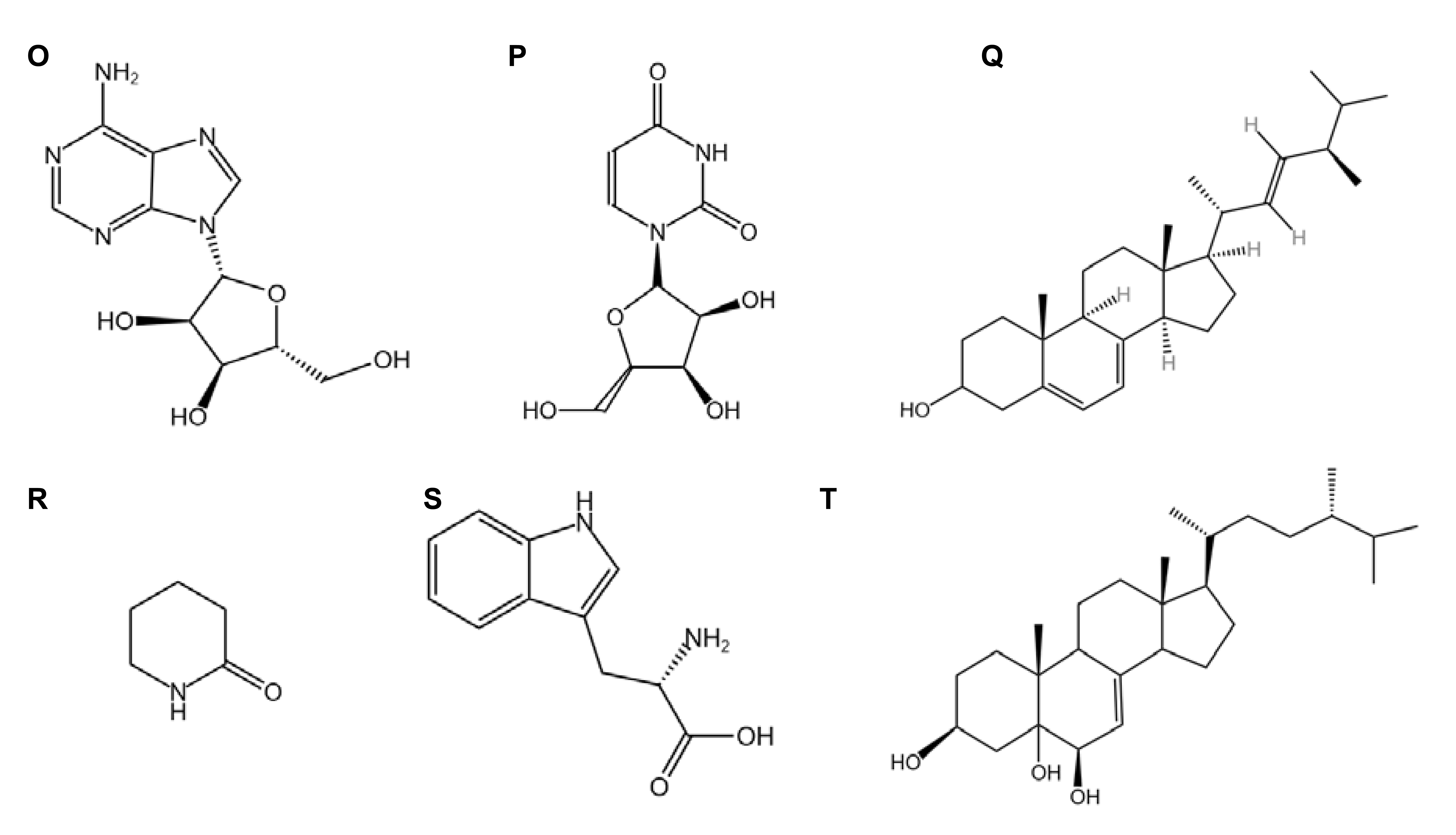
Supplementary Figure 1.** ^1^H NMR and ^13^C NMR spectra of the compounds extracted and obtained from *Ophiocordyceps sinensis* fruiting bodies. A and B: ^1^H NMR and ^13^C NMR spectra of cerevisterol; C and D: ^1^H NMR and ^13^C NMR spectra of adenosine; E and F: ^1^H NMR and ^13^C NMR spectra of uridine; G and H: ^1^H NMR and ^13^C NMR spectra of ergosterol; I and J: ^1^H NMR and ^13^C NMR spectra of 2-piperidone; K and L: ^1^H NMR and ^13^C NMR spectra of L-tryptophan; M and N: ^1^H NMR and ^13^C NMR spectra of (24S)-ergost-7(8)-en-3β,5α,6β-triol; O, P, Q, R, S, and T correspond to the chemical structures of adenosine, uridine, ergosterol, 2-piperidone, L-tryptophan, and (24S)-ergost-7(8)-en-3β,5α,6β-triol, respectively.


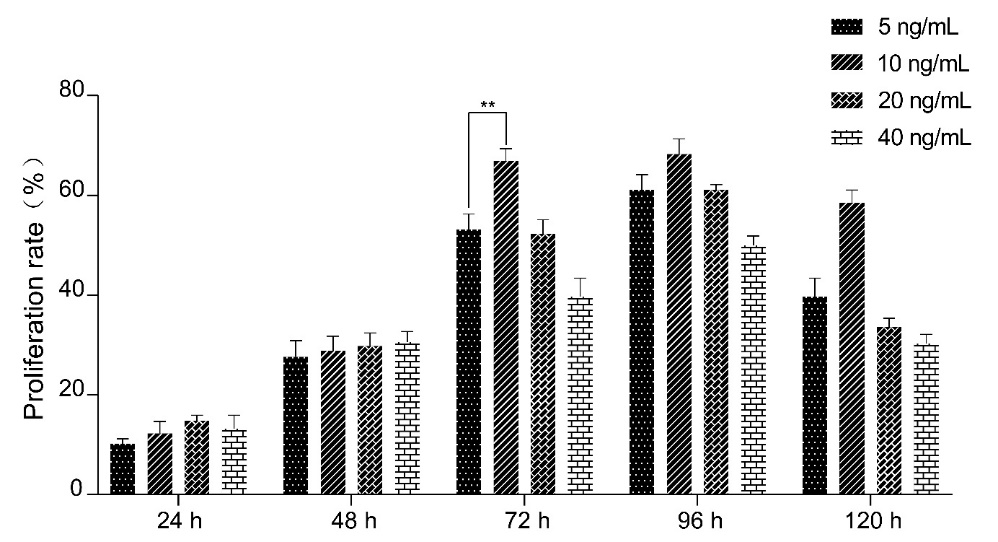


**Supplementary Figure 2.** Dose- and time-dependent effects of TGF-β1 on LX-2 cell viability. Cells were treated with TGF-β1 at concentrations of 5, 10, 20, and 40 ng/mL for 24, 48, 72, 96, and 120 h, respectively. Data are presented as mean ± SD (n = 3); **, significant difference at P < 0.01.


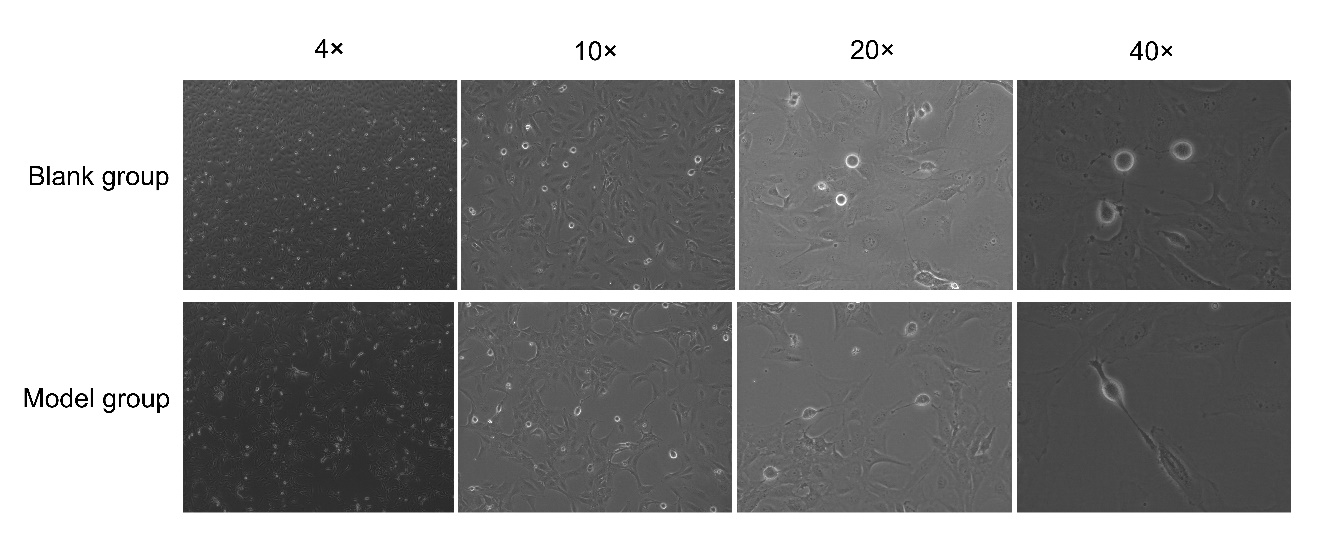


**Supplementary Figure 3.** Morphological changes in LX-2 cells following TGF-β1-induced activation: comparison between untreated control cells (Blank group) and cells cultured with TGF-β1 (10 ng/mL) for 72 h (Model group).
